# Formation and Inhibition of Heterocyclic Amines and Polycyclic Aromatic Hydrocarbons in Ground Pork during Marinating

**DOI:** 10.3390/foods11193080

**Published:** 2022-10-04

**Authors:** Yu-Wen Lai, Yu-Tsung Lee, Baskaran Stephen Inbaraj, Bing-Huei Chen

**Affiliations:** 1 Department of Food Science, Fu Jen Catholic University, New Taipei City 24205, Taiwan; 2 Research Center for Food and Cosmetic Safety, College of Human Ecology, Chang Gung University of Science and Technology, Taoyuan 33303, Taiwan; 3 Department of Nutrition, China Medical University, Taichung 40402, Taiwan

**Keywords:** heterocyclic amines, polycyclic aromatic hydrocarbons, marinated pork, UPLC-MS/MS, GC-MS/MS, QuEChERS, principle component analysis

## Abstract

This study aims to simultaneously extract heterocyclic amines (HAs) and polycyclic aromatic hydrocarbons (PAHs) from ground pork for respective analysis by UPLC-MS/MS and GC-MS/MS, and study the effects of different flavorings and marinating time length on their formation and inhibition. Results showed that both HA and PAH contents followed a time-dependent increase during marinating, with HAs being more susceptible to formation than PAHs. The total HA contents in unmarinated pork and juice was, respectively, 61.58 and 139.26 ng/g, and rose to 2986.46 and 1792.07 ng/g after 24-h marinating, which can be attributed to the elevation of reducing sugar and creatinine contents. The total PAH contents in unmarinated pork and juice were, respectively, 34.56 and 26.84 ng/g, and increased to 55.93 and 44.16 ng/g after 24-h marinating, which can be due to the increment of PAH precursors such as benzaldehyde, 2-cyclohexene-1-one and trans,trans-2,4-decadienal. Incorporation of 0.5% (w/v) cinnamon powder or 0.5% (w/v) green tea powder was effective in inhibiting HA formation with the former showing a more pronounced effect for marinated pork, while the latter was for marinated juice. However, their addition was only effective in inhibiting PAH formation in marinated pork. Principle component analysis revealed the relationship between HA and PAH formation in ground pork and juice during marinating.

## 1. Introduction

Both heterocyclic amines (HAs) and polycyclic aromatic hydrocarbons (PAHs) are vital toxic compounds mainly present in cooked protein-rich foods, especially meat products [1,2]. The formation mechanism of HAs and PAHs in meat/meat product during heating has been well established, with the former being generated through degradation of amino acid or protein during heating (>300 °C), or heating of amino acid, reducing sugar (6-carbon sugar) and creatine/creatinine at 100–300 °C, and the latter was produced through incomplete combustion or pyrolysis of organic materials such as fat, protein and carbohydrate at >200 °C [1,3,4]. Specifically, the Maillard reaction between amino acid and 6-carbon reducing sugar can lead to formation of α, β-dicarbonyl compound for the subsequent generation of α-amino-carbonyl compound through Strecker degradation. Next, pyridine or pyrazine can be formed through cyclization for the subsequent formation of imidazole through reaction with aldehyde and creatine/creatinine, followed by reaction with quinoline or quinoxaline to generate imidazoquinoline (IQ type HAs) and imidazoquinoxaline (IQx type HAs). While for pyrolytic HAs, both Glu-P-1 and Glu-P-2 can be produced from glutamic acid, Lys-P-1 from lysine, Phe-P-1 from phenylalanine as well as Trp-P-1, Trp-P-2, Norharman and Harman from tryptophan [5,6]. For PAHs, the benzene-containing compounds such as benzaldehyde has been shown to be a vital precursor for PAH formation through reaction with 1,3-butadiene, a degradation product from linoleic acid oxidation during heating for the subsequent Diels–Alder cycloaddition reaction [7]. According to International Agency of Research on Cancer [8], the HA IQ was classified as Group 2 A (probably carcinogenic) and PhIP, MeIQ, 8-MeIQx, AαC, MeAαC, Glu-P-1, Glu-P-2, Trp-P-1 and Trp-P-2 classified as Group 2 B (possibly carcinogenic) [9,10,11], while Norharman and Harman are generally considered as co-mutagen [12]. For PAHs, BaP was classified as Group 1 (carcinogenic); DBahA, CcdP and DBalP classified as Group 2 A; and NaP, CHR, BaA, MCH, BbFL, BkFL, BjF, IP, DBaiP and DBahP classified as Group 2 B [13]. As many HAs and PAHs possess potential carcinogenicity, it is urgent to learn the variety and amount of HAs and PAHs in various processed meat products.

The formation and inhibition of HAs and PAHs in meat/meat products during processing has been extensively studied. For instance, Hsu et al. [14] studied the effect of processing methods on HAs formation in duck meat and reported that both traditional oven and fan oven produced higher total HAs than the superheated steam oven, while the chicken fiber fried in lard at 180 °C/20 min was shown to generate a higher level of total HAs than in fried soybean oil [14]. In another study, Chen et al. [15] studied the effect of sugar smoking on PAH formation in meat, the level of total PAHs was found to be higher in red meat than in poultry meat, and the highly toxic BaP remained undetected in sugar-smoked meat. More recently, in a study dealing with formation of PAHs in thin slices of dried pork during roasting, soy sauce was found to be more efficient than sugar in reducing PAH formation; probably due to presence of isoflavone in the former [2]. Apparently, the formation of HAs and PAHs in meat/meat products can be affected by many factors such as meat variety, cooking time/temperature, cooking method, variety and amount of flavorings and cooking oil [4]. Thus, the inhibition of HAs and PAHs in meat products by added flavorings needs to be further investigated.

In recent years, a variety of spices/herbs and fruit extracts as well as phenolic-rich plant extracts and beverages were incorporated during meat marinating to study their inhibitory effects on formation of HAs and PAHs. By adding 0.5% of Sichuan pepper and 0.01% of sanshoamide extract, a total of HA inhibition by 70% was shown by Zeng et al. [16], while the incorporation of turmeric, curry leaf, torch ginger and lemon grass alone or in combination was reported to inhibit total HA contents ranging from 21.2 to 94.7%. In another study, Lu et al. [17] added 0.5% of garlic, onion, red chilli, paprika, ginger and black pepper powder to investigate their inhibitory effects on formation of HAs and PAHs in fried beef and chicken meat balls, and demonstrated that all the spice powders significantly reduced the formation of total HAs but not PAHs, with the ginger powder showing the most pronounced effect. In two different studies, the effect of phenolic compounds in beer on the formation of PAHs in charcoal-grilled chicken wings and pork was investigated, and a 67% inhibition of eight PAHs (BaA, CHR, BbF, BkFL, BaP, DBahA, BghiP and IP) was shown for the former [18], while a 30–68% inhibition of the same eight PAHs depending on the beer variety shown for the latter [19], with both studies showing a significant correlation between phenolic compounds in beer, antioxidant activity and PAHs inhibition. However, a poor correlation was shown between the antioxidant activity of added sugarcane molasses extract and inhibition of HAs formation in deep-fried chicken wings [20], as well as the antioxidant activity of green tea phenolic compounds and inhibition of PAHs formation in charcoal-grilled chicken wings [21], implying a complex inhibition mechanism involved in the reduction of HAs and PAHs formation. To evaluate the inhibitory effects of fruit extracts on HAs inhibition in roasted yellow croaker fish, Li et al. [22] recently reported that 1% blueberry extract, acerola cherry extract and grape seed extract could reduce the total HAs content with blueberry extract exhibiting the highest inhibitory effect and the inhibition mechanism can be associated with antioxidant activity of fruit extracts, generation of HAs precursors and lipid/protein oxidation during meat processing. Thus, it is necessary to explore some other natural products in inhibiting the formation of HAs and PAHs in meat products during processing.

Most of the reported studies discussed above were found to determine only a decreasing number of HAs or PAHs and there is a paucity of data dealing with comprehensive evaluation of most HAs and PAHs with simultaneous extraction of both. Nevertheless, in our recent study, Lai et al. [23] reported that by employing a QuEChERS (quick, easy, cheap, effective, rugged and safe) method, along with an optimal solvent system for extraction, a high accuracy and precision was attained for the simultaneous extraction of 20 HAs and 23 PAHs from pork jerky for the subsequent analysis by ultra-high-performance liquid chromatograph-tandem mass spectrometer (UPLC-MS/MS) and gas chromatograph-tandem mass spectrometer (GC-MS/MS), respectively. Thus, this study aims to adopt the above QuEChERS method for the simultaneous extraction of HAs and PAHs from marinated pork and juice, a popular meat commodity in Taiwan, for further analysis by UPLC-MS/MS and GC-MS/MS, and explore the effects of marinating time length and different flavorings including soy sauce, sugar, cinnamon powder and green tea powder on formation and inhibition of HAs and PAHs in ground pork/juice during marinating. 

## 2. Materials and Methods

### 2.1. Processing of Marinated Pork

A total of 9 kg of raw ground pork was divided into 18 groups with 500 g each for marinating. The unmarinated juice was prepared by mixing of 10 g (1%) of sugar, 100 g (10%) of soy sauce and 890 g (89%) of deionized water (standard formula). In addition, two more formulas were prepared: standard formula plus 0.5% (weight/volume, w/v) cinnamon powder and standard formula plus 0.5% (w/v) green tea powder. Each unmarinated juice was poured into a stainless-steel saucepan with a lid on the top and heated by gas. After boiling, 500 g of raw ground pork was poured into a saucepan and heated for 2, 4, 8, 12 and 24 h, and a total of 18 saucepans, including 3 saucepans without heating, were used. The temperature was controlled at 90 ± 2 °C, and 200 mL of hot water added to maintain the volume at 1 L every hour during marinating. Before sampling, water was added to the same original volume as that before heating. Then the marinated pork and juice were subjected to analysis of HAs and PAHs by QuEChERS coupled with UPLC-MS/MS and GC-MS/MS, respectively. 

### 2.2. Materials and Chemical Reagents

A total of 9 kg of raw ground pork was purchased from a local supermarket located in New Taipei City, Taiwan, and was stored in a −20 °C freezer prior to use. A total of 21 HA standards including DMIP, IFP, iso-IQ, IQ, MeIQ, IQ [4,5-b], IQx, 8-MeIQx, 7,8-DiMeIQx, 4,8-DiMeIQx, 4,7,8-TriMeIQx (internal standard), Phe-P-1, AαC, Trp-P-2, Trp-P-1, GIu-P-2, Glu-P-1, PhIP, Harman, Norharman and MeAαC were procured from Toronto Research Chemicals (Downsview, Ontario, Canada), while 24 PAH standards including Nap, AcPy, AcP, Flu, Phe, Ant, FL, Pyr, BaA, CHR, BbFL, BaP, IP, DBahA, BghiP, BjF, BcF, CcdP, MCH, DBalP, DBaeP, DBaiP, DBahP and Triphenylene (internal standard) were from Sigma-Aldrich Co. (St. Louis, MO, USA). A list of names and abbreviations of all the 21 HAs and 24 PAHs used in this study are provided at the end of this article. 

The HPLC-grade solvents including methanol, acetonitrile, hexane and acetone were from Merck Co. (Darmstadt, Germany), while glacial acetic acid was from Sigma-Aldrich Co. Deionized water was made using a Milli-Q water purification system from Millipore Co. (Bedford, MA, USA). An Acquity UPLC BEH C18 column (100 × 2.1 mm ID., particle size 1.7 μm) was from Waters Co. (Milford, MA, USA), and a DB-5 MS capillary column (30 m × 0.25 mm ID, film thickness 0.25 μm) was from Agilent Technologies Co. (Palo Alto, CA, USA). The QuEChERS extraction (UR-EX) and purification (UR-CLEAN-II) kit was from Yu-Ho Co. (New Taipei City, Taiwan).

### 2.3. Simultaneous Extraction and Purification of HAs and PAHs in Unmarinated/Marinated Pork and Juice by QuEChERS 

A method based on Lai et al. [23] was used for the simultaneous extraction of HAs and PAHs from marinated pork and juice. In brief, a 2-g sample of unmarinated pork, marinated pork or juice was mixed with one ceramic homogenizer in a 50 mL centrifuge tube, followed by adding 10 mL of deionized water and shaking the mixture at 200 rpm for 10 min. Then 10 mL of 100% acetonitrile containing 1% acetic acid was added for shaking at 200 rpm for 10 min, after which the extraction powder (MgSO_4_ 4 g and CH_3_ COONa 1 g) was added for shaking for one min, followed by centrifuging at 4000× *g* for 10 min (4 °C) and collecting the supernatant (6 mL) for purification. Then the supernatant was poured into a centrifuge tube containing 300 mg PSA, 900 mg MgSO_4_ and 300 mg C18 EC for shaking for 1 min, and then centrifuged at 4000× *g* for 10 min (4 °C). Then the supernatant (1 mL) was collected, evaporated to dryness under nitrogen, dissolved in 0.2 mL of methanol containing 1 ppb of internal standard (4,7,8-TriMeIQx) and filtered through a 0.22-μm membrane filter for HA analysis by UPLC-MS/MS. For PAH analysis, the residue was dissolved in 0.2-mL of hexane containing 10 ppb of internal standard (Triphenylene) and filtered through a 0.22-μm membrane filter for GC-MS/MS analysis.

### 2.4. Separation and Identification of HAs in Unmarinated/Marinated Pork and Juice by UPLC-MS/MS

A method based on Hsu and Chen [1] was used to analyze HAs in unmarinated pork, marinated pork and juice by UPLC-MS/MS. An ACQUITY BEH C18 column (50 × 2.1 mm ID, particle size 1.7 μm) and a gradient mobile phase of (A) 20 mM ammonium acetate (pH 4.75) and (B) 100% acetonitrile with an initial proportion of 95% A and 5% B, maintained for 2 min, raised to 90% B in 3.3 min, maintained for 4 min and returned to original ratio in 4.1 min for separation of 21 HAs with the column temperature at 30 °C and flow rate at 0.7 mL/min. The various MS parameters and selected reaction monitoring (SRM) mode used for detection of various HAs in unmarinated/marinated pork and juice as well as precursor ions and product ions were the same as those reported by Hsu and Chen [24].

### 2.5. Separation and Identification of PAHs in Unmarinated/Marinated Pork and Juice by GC-MS/MS

A method based on Lai et al. [23] was used to analyze PAHs in unmarinated pork, marinated pork and juice. A DB-5 MS capillary column (30 m × 0.25 mm ID, film thickness 0.25 μm) with He as carrier gas, flow rate at 1.25 mL/min, injector temperature at 320 °C, injection in splitless mode and MS interface temperature at 280 °C was used to separate 23 PAHs within 78 min with the following temperature programming condition: 80 °C initially, maintained for 1 min, increased to 200 °C at 5 °C/min, maintained for 10 min, raised to 220 °C at 5 °C/min, maintained for 5 min, increased to 230 °C at 1 °C/min, maintained for 10 min, and raised to 320 °C at 10 °C/min and maintained for 10 min.

The SRM mode was used for detection of various PAHs in unmarinated pork, marinated pork and juice samples, and the parameters, such as precursor ions and product ions, were the same as that reported by Lai et al. [23]. Furthermore, an internal standard (Triphenylene) was added to pork sample for quantitation of various PAHs in unmarinated/marinated pork and juice.

### 2.6. Method Validation of HAs and PAHs

The method validation of HAs and PAHs in freeze-dried raw ground pork was reported in our recent study [23], and the same raw ground pork was used to prepare the marinated pork in this study. Thus, the method validation was not performed separately in this study.

### 2.7. Determination of HA Precursors

The determination of HA precursors such as reducing sugar, amino acid and creatine/creatinine in unmarinated pork/juice as well as marinated pork/juice was performed by a method reported by Chen et al. [24], TFDA [25] and Gibis and Loeffler [26], respectively. A detailed procedure is provided in the Supplementary Material.

### 2.8. Determination of Antioxidant Activity

The determination of antioxidant activity of unmarinated pork/juice as well as marinated pork/juice was performed by a method reported by Serpen et al. [27]. A detailed procedure is provided in the Supplementary Material. 

### 2.9. Determination of Bioactive Compounds in Cinnamon Powder by UPLC-MS/MS

The determination of bioactive compounds in cinnamon powder was performed by a method reported by Wang et al. [28]. A detailed procedure is provided in the Supplementary Material.

### 2.10. Determination of Bioactive Compounds in Green Tea Powder by HPLC-Diode Array Detection (DAD)

The determination of bioactive compounds in green tea powder was performed by a method reported by Lin et al. [29]. A detailed procedure is provided in the Supplementary Material.

### 2.11. Determination of PAH Precursors

The determination of PAH precursors of unmarinated pork/juice as well as marinated pork/juice was performed by a method reported by Bueno et al. [30]. A detailed procedure is provided in the Supplementary Material.

### 2.12. Principal Component Analysis (PCA)

Principal component analysis (PCA) was performed on the mean data of triplicate determinations by grouping experimental data of various treatments and transforming a set of correlated variables into a new set of linearly uncorrelated variables based on eigenvalue >1. The possible correlation between formation of HAs and PAHs in marinated pork/juice as affected by different flavorings and marinating time length were elucidated by running PCA with a Kaiser–Meyer–Olkin value of 0.80 and *p* < 0.05 by using Origin^®^ 2019 b version 9.65 (OriginLab Corporation, Northampton, MA, USA).

### 2.13. Statistical Analysis

All the data were subjected to statistical analysis using the statistical analysis system (SAS) 9.4 software system [31] for analysis of variance (ANOVA) and Duncan’s multiple range test for significance in mean comparison (*p* < 0.05).

## 3. Results and Discussion

### 3.1. HA Contents in Unmarinated/Marinated Pork and Juice as Affected by Flavorings and Heating Time 

**Figure 1:**
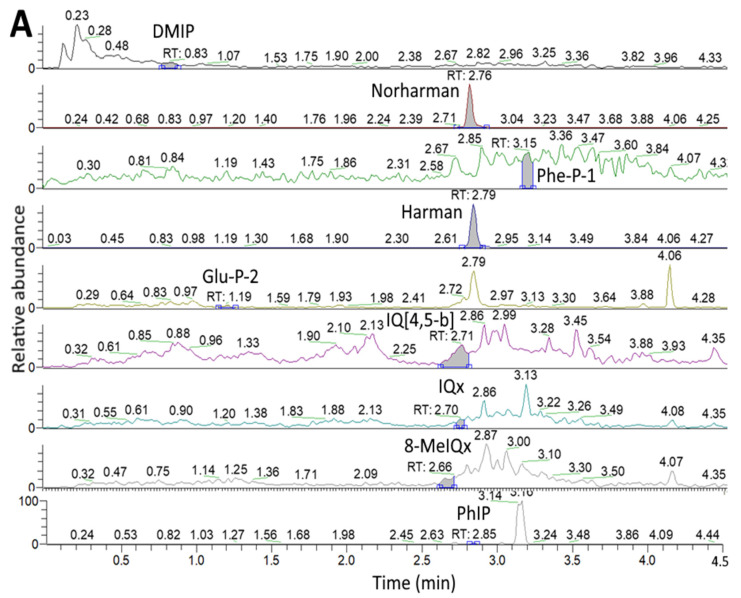

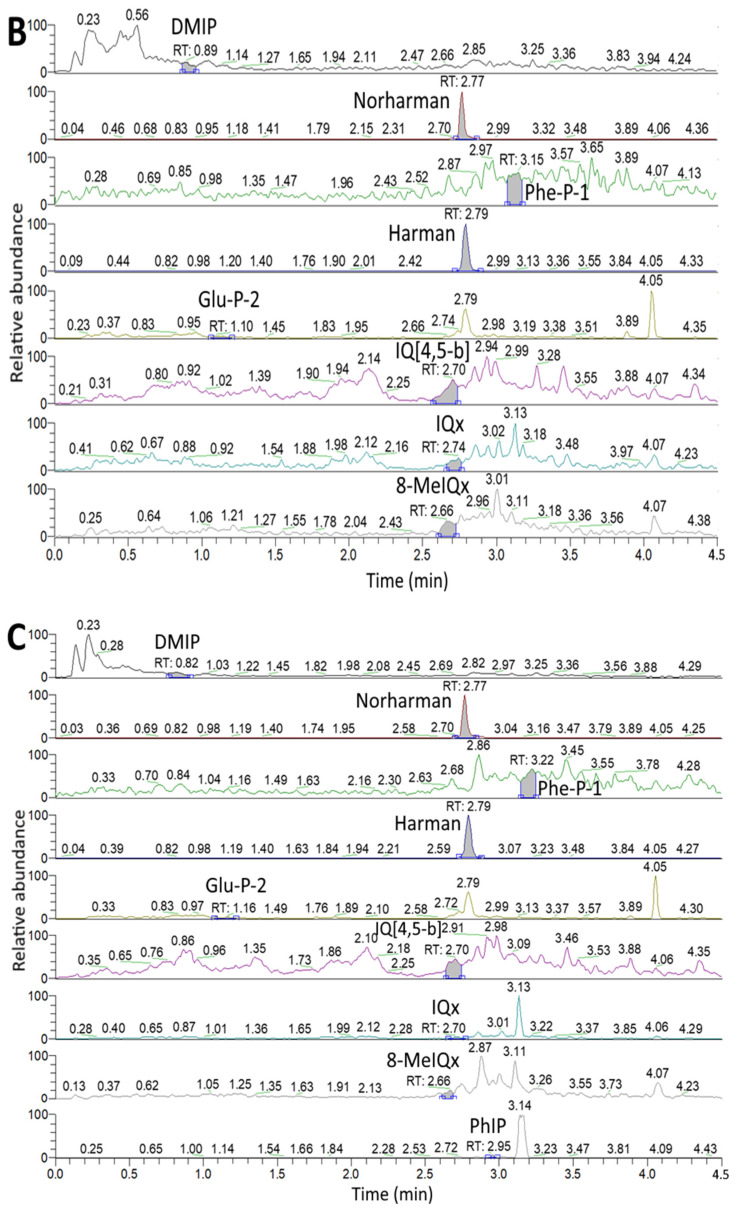
UPLC-MS/MS chromatogram of HAs in marinated pork after 24 h marinating (**A**), marinated pork with 0.5% cinnamon powder after 24 h marinating (**B**) and marinated pork with 0.5% green tea powder after 24 h marinating (**C**), as detected by SRM mode.

**Figure 2:**
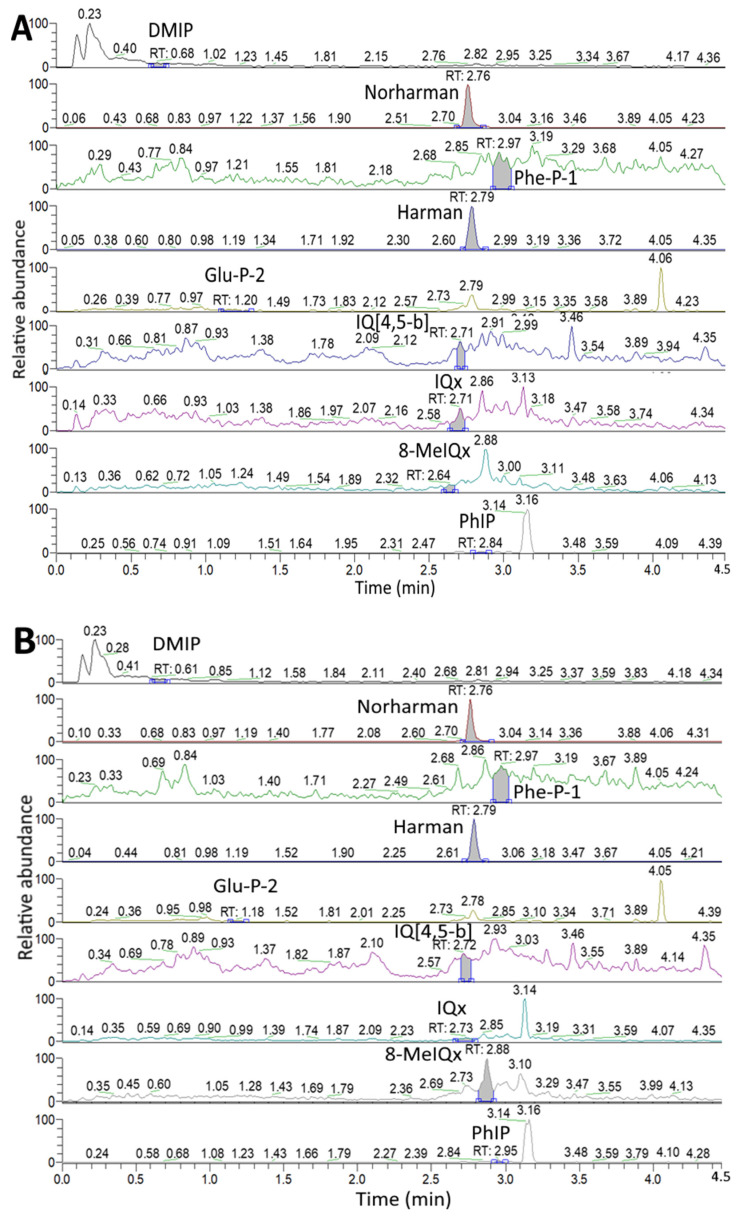

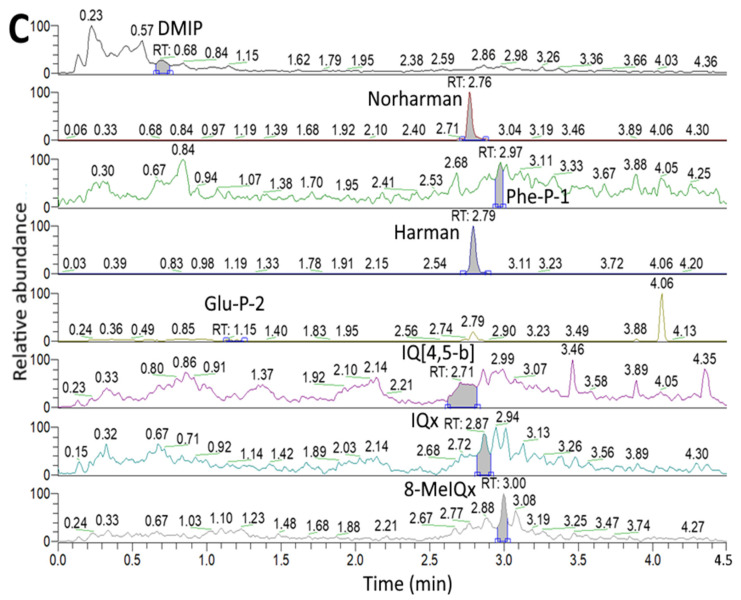
UPLC-MS/MS chromatogram of HAs in marinated juice (**A**), marinated juice with 0.5% cinnamon powder after 24 h marinating (**B**) and marinated juice with 0.5% green tea powder after 24 h marinating (**C**), as detected by SRM mode.

Figure 1 and Figure 2 show the UPLC-MS/MS chromatogram of HAs, respectively, in marinated pork and juice after 24 h marinating without and with 0.5% cinnamon powder or 0.5% green tea powder, as detected by SRM mode. The HA contents in unmarinated/marinated pork and juice as affected by flavorings and heating time are shown in Table 1. A total of three HAs including Harman (53.51 ng/g), Norharman (7.92 ng/g) and Glu-P-2 (0.15 ng/g) were present in unmarinated pork (Table 1), while a total of four HAs including Harman (120.42 ng/g), Norharman (16.13 ng/g), Phe-P-1 (2.41 ng/g) and Glu-P-2 (0.30 ng/g) were present in unmarinated juice (Table 1). Apparently, the unmarinated juice contained a higher level of total HAs than that in unmarinated pork. The presence of high level of Harman and Norharman in both unmarinated pork and juice is probably from soy sauce as it was reported that amino acids such as tryptophan may react with pyruvate or acetate to generate Harman and Norharman [32]. In a previous study, Pfau and Skog [32] pointed out that Harman and Norharman were present at a level of 130–250 ng/mL and 5–71 ng/mL in soy sauce, respectively. Moreover, the contents of Harman and Norharman in five commercial soy sauces were shown to be from 111.47–301.30 ng/g and 80.76–199.27 ng/g, respectively [33].

In Table 1, the HA contents were shown to follow a time-dependent rise during pork marinating, and a total of nine HAs were detected after 24-h marinating, with Harman present at the highest level (2456.96 ng/g), followed by Norharman (335.49 ng/g), Phe-P-1 (189.12 ng/g), IQ [4,5-b] (1.53 ng/g), Glu-P-2 (1.01 ng/g), PhIP (0.95 ng/g), DMIP (0.86 ng/g), IQx (0.47 ng/g) and 8-MeIQx (0.10 ng/g). However, after incorporation of 0.5% cinnamon powder or 0.5% green tea powder, the total HA contents were reduced to 2641.90 and 2528.75 ng/g, respectively, following 24-h marinating, implying that green tea powder was more effective than cinnamon powder in minimizing HA formation in marinated pork.

Like marinated pork, the HA contents also followed a time-dependent increase during juice marinating. Following 24-h marinating, a total of nine HAs were produced with Harman present at the highest level (1481.22 ng/g), followed by Norharman (204.82 ng/g), Phe-P-1 (102.72 ng/g), DMIP (1.33 ng/g), Glu-P-2 (0.96 ng/g), PhIP (0.48 ng/g), IQx (0.37 ng/g), 8-MeIQx (0.18 ng/g) and IQ [4,5-b] (trace) (Table 1). However, following the addition of 0.5% cinnamon powder or 0.5% green tea powder, the total HA contents were reduced to 1282.32 and 1398.56 ng/g, respectively, after 24-h marinating, revealing that cinnamon powder was more efficient than green tea powder in minimizing HA formation in marinated juice. Furthermore, in both marinated pork and juice, the non-polar HAs such as Harman, Norharman, Phe-P-1 and Glu-P-2 were more susceptible to formation than the polar HAs during marinating. This outcome is in accordance with a report by Gibis [34], showing that the non-polar HAs were more readily formed at a lower temperature. As both Harman and Norharman were shown to enhance mutagenicity of other toxic compounds such as Trp-p-1, BaP, N-2-fluorenylacetamide and 4-dimethyl aminoazobenzene [35], their presence at high levels in marinated pork and juice cannot be ignored.

#### 3.1.1. Amino Acid Change in Unmarinated/Marinated Pork and Juice

Table 2 shows changes of individual and total amino acid contents in unmarinated/marinated pork and juice. Only a minor change in total amino acid contents was shown over a 24-h marinating period for marinated pork, marinated pork plus 0.5% cinnamon powder and marinated pork plus 0.5% green tea powder, indicating that amino acid may play a less important role in HA formation. A similar phenomenon was found for the treatments of marinated juice, marinated juice plus 0.5% cinnamon powder and marinated juice plus 0.5% green tea powder (Table 2).

#### 3.1.2. Reducing Sugar Content Change in Unmarinated/Marinated Pork and Juice

Conversely, reducing sugar should play a more important role than amino acid in HA formation in marinated pork, marinated pork plus 0.5% cinnamon powder and marinated pork plus 0.5% green tea powder, as evidenced by a time-dependent rise in reducing sugar content being shown for all the treatments during pork marinating (Table 3). Specifically, following 24-h marinating, the reducing sugar content was raised from 0.09 mg/g in unmarinated pork to 4.03 mg/g in marinated pork. Similarly, after addition of 0.5% cinnamon powder or 0.5% green tea powder, the reducing sugar contents further increased to 5.33 mg/g and 6.50 mg/g, respectively, following 24-h marinating, which can be attributed to the decomposition of sucrose into glucose and fructose during heating. Compared to marinated pork, a higher level of reducing sugar in the treatments of 0.5% cinnamon powder and 0.5% green tea powder implied that a less amount of reducing sugar participated in the Maillard browning reaction, leading to a reduction in total HAs (Table 1). However, a different trend in reducing sugar content change was shown in marinated juice (Table 3), as a plateau was reached after 2-h marinating for marinated juice (12.18 mg/g), marinated juice plus 0.5% cinnamon powder (11.01 mg/g) and marinated juice plus 0.5% green tea powder (8.92 mg/g), followed by a decline to 7.89, 9.74 and 8.57 mg/g, respectively, after 24-h marinating. It may be postulated that the decrease in reducing sugar content during marinating is probably due to the reaction rate of Maillard browning reaction being higher than the decomposition rate of sucrose. Compared to 0.5% green tea powder, the addition of 0.5% cinnamon powder resulted in a more reduction of the reducing sugar content during 24-h marinating, revealing that a higher amount of reducing sugar participated in the Maillard browning reaction, leading to a further reduction of total HAs for the latter treatment (Table 1).

#### 3.1.3. Creatine and Creatinine Change in Unmarinated/Marinated Pork and Juice

Creatine, commonly present in creatine phosphate form in animal muscle tissue as energy source, can be hydrolyzed to creatinine during heating, a precursor of imidazole formation [36,37]. Moreover, the formation of creatine/creatinine in meat products during heating can be correlated positively with mutagenicity [38]. In Table 3, the creatine content followed a time-dependent decrease in marinated pork, marinated pork plus 0.5% cinnamon powder and marinated pork plus 0.5% green tea powder and declined to 6.72, 7.27 and 7.62 mg/100 g after 24-h marinating, respectively, which can be attributed to the conversion of creatine to creatinine during heating. A similar trend was observed for the creatine content change in marinated juice, marinated juice plus 0.5% cinnamon powder and marinated juice plus 0.5% green tea powder, with the creatine content being reduced to 9.30, 10.26 and 10.79 mg/100 g, respectively, after 24-h marinating (Table 3). Comparatively, unmarinated pork contained a much higher level of creatine than unmarinated juice, however, following marinating for 4 h and above, marinated juice was shown to contain a higher level of creatine than marinated pork, which may partially due to leaching of creatine from pork into juice. Both treatments of 0.5% cinnamon powder and 0.5% green tea powder showed the same phenomenon. For the creatinine content change, it followed a time-dependent rise over a 8-h marinating period and a maximum was reached for marinated pork (76.43 mg/100 g), marinated pork plus 0.5% cinnamon powder (56.60 mg/100 g) and marinated pork plus 0.5% green tea powder (51.65 mg/100 g), followed by a decline to 44.47, 45.43 and 49.31 mg/100 g, respectively, after 24-h marinating (Table 3), probably due to participation of creatinine in the Maillard browning reaction. It is also possible that the formation rate of creatinine from creatine was slower than the Maillard browning reaction rate after prolonged marinating for ≥8 h.

A similar trend was observed for the creatinine content change in marinated juice, marinated juice plus 0.5% cinnamon powder and marinated juice plus 0.5% green tea powder, with a peak of 14.44, 10.74 and 9.76 mg/100 g being attained, respectively, after 8-h marinating, accompanied by a drop to 8.20, 8.29 and 9.37 mg/100 g, respectively, after prolonged marinating for 24 h (Table 3). Like marinated pork, the formation rate of creatinine from creatine should be slower than the Maillard browning reaction rate for all the three marinated juice treatments.

#### 3.1.4. Antioxidant Capacity of Unmarinated/Marinated Pork and Juice

Table 4 shows the antioxidant capacity of unmarinated pork, marinated pork and juice. Interestingly, only a minor change in DPPH value was shown for both marinated pork and juice over a 24-h marinating period. However, for FRAP, both marinated pork and marinated pork plus 0.5% cinnamon powder followed a time-dependent increase during marinating for 24 h. For the treatments of marinated juice and marinated pork plus 0.5% green tea powder, the highest FRAP was observed after 8-h marinating, followed by a decline afterwards. Nevertheless, the addition of 0.5% cinnamon powder or 0.5% green tea powder was shown to increase FRAP in both marinated pork and juice, which can be attributed to the presence of high level of cinnamon aldehyde (2.34 mg/g) in cinnamon powder and total catechin (114.64 mg/g) in green tea powder (Table 5). 

In several previous studies, Unal et al. [39] reported that the addition of 0.2% clove or 0.5% cinnamon was effective in inhibiting HA formation in barbecued sucuk, while the HA formation in meat and seafood was retarded by incorporation of cinnamon aldehyde [40]. Likewise, the incorporation of 0.5% garlic, onion, red chilli, paprika, ginger or black pepper was shown to reduce total HAs content, but not PAHs in beef and chicken meatballs fried at 180 °C, and a high negative correlation was shown between TEAC/ORAC antioxidant activity of these spices and total HAs content [17]. By using an optimum ratio of turmeric to lemon grass at 52.4:47.6, Sepahpour et al. [41] demonstrated a 94.7% inhibition of total HAs content in grilled beef with a reduction of IQ, PhIP, Harman, Norharman and AαC by 52, 92, 99, 97 and 98%, respectively, while an inhibition of 82, 61, 28 and 79% was shown for PhIP, IQx, MeIQx and 4,8-DiMeIQx, respectively, in grilled beef marinated with 0.5% Sichaun pepper and 0.01% sanshoamide extract [16]. In a later study, Teng et al. [42] explored the inhibitory effects of *Sonchus olearleu* herb extract on HAs formation in roasted pork patties and reported that a significant reduction in IQ, Harman and Norharman was correlated negatively with total phenolic content and antioxidant potential of *S. olearleu* herb extract. More recently, Lai et al. [23] demonstrated that the addition of 0.5% cinnamon powder was efficient in inhibiting formation in pork jerky during roasting. Similar findings were shown for HA inhibition in pan-fried beef marinated with green tea, and the longer the marinating time, the less the formation of HAs in beef during pan-frying [43]. In addition to the antioxidant capacity of cinnamon powder and green tea powder, the effect of Maillard browning reaction products (MRPs) on inhibition of HAs cannot be ignored. As shown in Table 4, the Maillard browning reaction index followed a time-dependent rise for all three marinated juice treatments, with green tea powder showing a higher Maillard browning reaction index than cinnamon powder and marinated juice after prolonged marinating, leading to a reduction in total HAs in marinated juice (Table 1). A similar phenomenon was observed by Lai et al. [23], reporting that the formation of MRPs was effective in inhibiting HA formation in roasted pork jerky. 

Taken together, the addition of 0.5% cinnamon powder was more effective than 0.5% green tea powder in inhibiting HA formation in marinated pork, while in marinated juice, the incorporation of 0.5% green tea powder was more efficient than 0.5% cinnamon powder. This difference may be accounted for by the presence of the low-polar cinnamon aldehyde from cinnamon powder mainly in marinated pork, while the polar catechin in green tea powder may be mainly present in marinated juice.

### 3.2. Analysis of PAHs in Unmarinated/Marinated Pork and Juice

**Figure 3:**
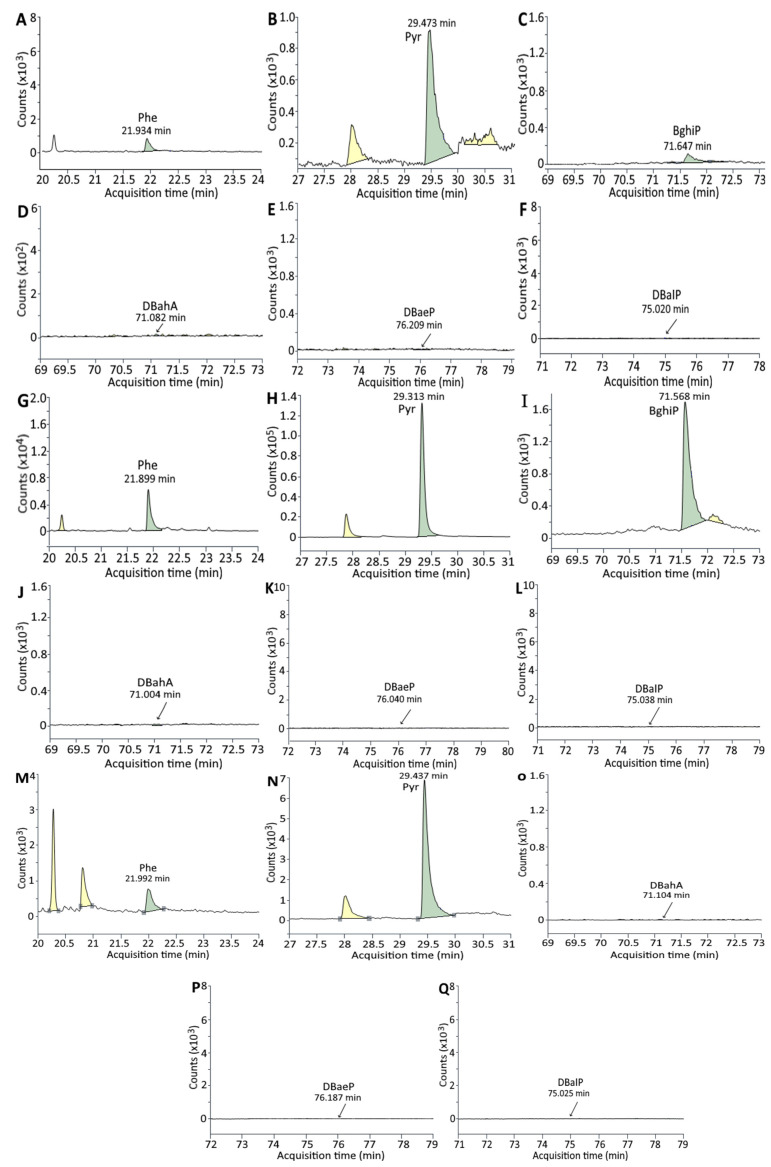
GC-MS/MS chromatogram of PAHs in marinated pork after 24 h marinating (**A**–**F**), marinated pork with 0.5% cinnamon powder after 24 h marinating (**G**–**L**) and marinated pork with 0.5% green tea powder after 24 h marinating (**M**–**Q**), as detected by SRM mode. (**A**,**G**,**M**): Phe; (**B**,**H**,**N**): Pyr; (**C**,**I**): BghiP; (**D**,**J**,**O**): DBahA; (**E**,**K**,**P**): DBaeP; (**F**,**L**,**Q**): DBalP.

**Figure 4:**
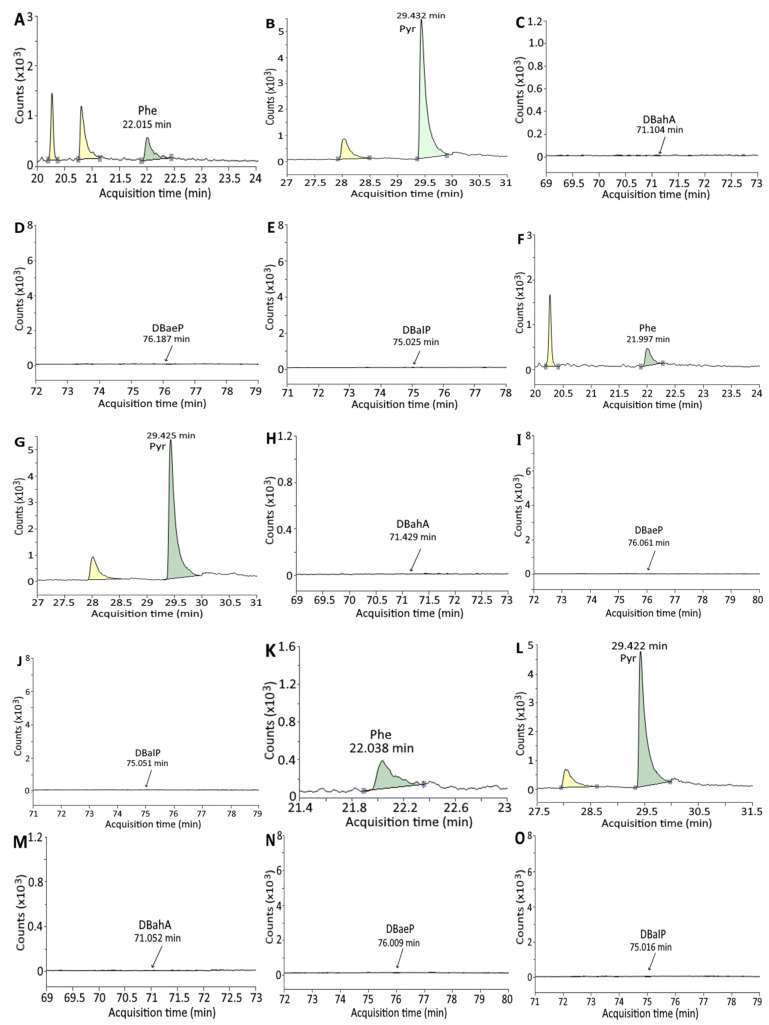
GC-MS/MS chromatogram of PAHs in marinated juice after 24 h marinating (**A**–**E**), marinated juice with 0.5% cinnamon powder after 24 h marinating (**F**–**J**) and marinated juice with 0.5% green tea powder after 24 h marinating (**K**–**O**), as detected by SRM mode. (**A**,**F**,**K**): Phe; (**B**,**G**,**L**): Pyr; (**C**,**H**,**M**): DBahA; (**D**,**I**,**N**): DBaeP; (**E**,**J**,**O**): DBalP.

Figure 3 and Figure 4 show the GC-MS/MS chromatogram of PAHs in marinated pork and juice, respectively, after 24 h marinating without and with addition of 0.5% cinnamon powder or 0.5% green tea powder. The PAH contents in marinated pork as affected by time length are shown in Table 6. A total of five PAHs were found in unmarinated pork, with pyrene being present in the largest amount (31.00 ng/g), followed by DBahA (1.54 ng/g), DBalP (0.72 ng/g), DBaeP (0.71 ng/g) and Phe (0.60 ng/g). However, a time-dependent increase in PAH contents was shown during marinating for 24 h, with six PAHs being generated at the highest level including Pyr (50.51 ng/g), Phe (1.64 ng/g), DBahA (1.54 ng/g), BghiP (0.81 ng/g), DBalP (0.72 ng/g) and DBaeP (0.71 ng/g). The same tendency was observed for both treatments of marinated pork plus 0.5% cinnamon powder and marinated pork plus 0.5% green tea powder, with the total PAH contents of 58.76 and 58.04 ng/g being produced, respectively. By comparison at the same time length (2–12 h), the addition of 0.5% cinnamon powder or 0.5% green tea powder was effective in reducing PAH formation. However, after prolonged marinating for 24 h, the addition of 0.5% cinnamon powder or 0.5% green tea powder failed to reduce PAH formation, probably due to degradation of bioactive compounds such as cinnamaldehyde in cinnamon powder or catechin in green tea powder.

Similar to unmarinated pork, a total of five PAHs including Pyr (23.56 ng/g), DBahA (1.53 ng/g), DBalP (0.72 ng/g), DBaeP (0.71 ng/g) and Phe (0.34 ng/g) were detected in unmarinated juice (Table 6). A time-dependent rise in PAH contents was also shown over a 24-h marinating period, with Pyr present at the highest level (40.36 ng/g), followed by DBahA (1.55 ng/g), Phe (0.80 ng/g), DBalP (0.74 ng/g) and DBaeP (0.73 ng/g). Both treatments of marinated juice plus 0.5% cinnamon powder and marinated juice plus 0.5% green tea powder showed the same trend; however, by comparison at the same time length, the incorporation of 0.5% cinnamon powder generated the highest level of total PAHs (58.39 ng/g) followed by 0.5% green tea powder (51.68 ng/g) and marinated juice (44.17 ng/g). This outcome implied that both 0.5% cinnamon powder and 0.5% green tea powder failed to inhibit PAH formation in marinated juice, probably due to degradation of bioactive compounds during heating as indicated above. Nevertheless, the bioactive compounds in cinnamon powder and green tea powder may be more susceptible to degradation in marinated juice when compared to marinated pork. Furthermore, the presence of five PAHs in unmarinated juice may be originally from soy sauce as 3,4-benzopyrene was detected in Japanese soy sauce [44]. In a study dealing with the effect of curing on PAH formation in poultry meat, a small amount of PAHs were detected in raw meat, while the total PAH was produced at a higher level for 24-h curing than for 12-h curing [3]. This result is similar to our finding that a small amount of PAHs were present in both unmarinated pork and juice.

The PAH formation and inhibition in model system and food products during heating has been controversial. For instance, Britt et al. [45] studied PAH formation in three model systems by heating at 840 °C for 10 sec, a model system of glucose and proline was shown to generate the highest amount of PAHs (Phe and Ant), followed by the model systems of glucose and proline. Obviously, the formation of MRPs between glucose and proline during heating can promote PAH formation. In a study dealing with the effect of small molecular weight aldose and basic amino acid on PAH formation in grilled pork sausage, Nie et al. [46] pointed out that glucose was more susceptible to PAH formation than fructose, probably due to formation of small aldehyde compounds from the former for the subsequent polymerization or condensation to produce PAHs. Furthermore, the addition of basic amino acids such as lysine and arginine was shown to generate more PAHs than acidic amino acids, probably caused by a rise in pH from the former to accelerate Maillard reaction for the subsequent formation of 1-amino-1-deoxy-2-ketosaccharide and then PAHs [46]. More specifically, sugar can be dehydrated to form 5-HMF, methylglyoxal and acetone compounds under acidic condition, followed by indene formation from 5-HMF through Diels–Alder reaction, dehydrogenation and acetylene addition to generate FL [47,48,49]. In meat products, the PAH formation in thin slices of dried pork as affected by flavorings and roasting conditions was studied by Hung et al. [2], reporting that a flavoring of 16% sugar and 8% soy sauce or 16% sugar and 4% soy sauce generated some more varieties of PAHs at 200 °C. Furthermore, the AcP oxidation during roasting of thin slices of dried pork may lead to AcPy formation. In another study the addition of elderberry was shown to inhibit PAH formation in charcoal-grilled pork by 82%, followed by white wine vinegar (79%), red wine vinegar (66%), apple cider vinegar (66%) and fruit vinegar with raspberry juice (55%) [50]. Sinaga et al. [51] further reported that following marinating in andaliman fruit juice for 60 min for the subsequent grilling, the BaP content in charcoal-grilled duck was reduced by 2.6-fold. 

#### Analysis of PAH Precursors in Unmarinated/Marinated Pork and Juice

Table 7 shows the content changes of PAH precursors including 2-cyclohexene-1-one, benzaldehyde and trans,trans-2,4-decadienal in marinated pork and juice. In most treatments the total amount of PAH precursors followed a time-dependent increase and resulted in a rise of total PAH contents. Of the three precursors, only 2-cyclohexene-1-one and trans,trans-2,4-decadienal followed a time-dependent increment for all the marinated pork and juice treatments during marinating, while an inconsistent change was observed for the benzaldehyde, probably due to its volatility and instability. Comparatively, benzaldehyde was produced at a higher level than 2-cyclohexene-1-one and trans,trans-2,4-decadienal in marinated pork and juice, and should play a more important role in PAH formation as reported by Chen and Chen [7]. Furthermore, the addition of 0.5% cinnamon powder may promote benzaldehyde formation in both marinated pork and juice, as it was reported that cinnamaldehyde could be converted to benzaldehyde during heating [52]. This may explain why a maximum level of benzaldehyde (922.47 ng/g) was produced in pork after 2-h marinating. Moreover, the addition of 0.5% green tea powder may induce 2-cyclohexene-1-one formation as it was detected in green tea [53]. Conversely, the addition of 0.5% cinnamon powder or 0.5% green tea powder was effective in inhibiting formation of trans,trans-2,4-decadienal, a degradation product of linoleic acid oxidation during heating. In a previous study, Chen and Chen [7] studied the PAH formation mechanism in food lipids, and postulated that benzaldehyde could be generated through cyclohexene oxidation, while trans,trans-2,4-decadienal and 2-cyclohexene-1-one produced through degradation of linoleic acid and linolenic acid during heating, respectively.

The inhibition of PAHs in meat products by incorporation of antioxidants has been well documented. For instance, the addition of onion (30 g/100 g pork) or garlic (15 g/100 g pork) was effective in inhibiting six PAHs (BbF, BkFL, BaA, BaP, DBahA and BghiP) in fried pork by 60% and 54%, respectively [54,55]. Wang et al. [56] reported that 57% of eight PAHs including BaA, CHR, BbF, BkFL, BaP, DBahA, BghiP and IP in charcoal-grilled chicken wings was inhibited by green tea, which can be attributed to the presence of phenolic compounds such as catechin. In two different studies, the beer-marinated charcoal-grilled chicken wings were shown to reduce the formation of PAHs with both phenolic content and antioxidant activity of beer playing a significant role [18,19]. More recently, Cao et al. [57] compared the effect of apple polyphenol and three antioxidants (ascorbic acid, TBHQ and BHT) on PAH formation in barbecued pork and reported that the addition of 0.2% apple polyphenol possessed the most pronounced inhibition effect which correlated well with the antioxidant activity, peroxide and thiobarbituric acid values. For phenolic acids, protocatechuic acid was shown to be the most effective in inhibiting PAH formation in charcoal-grilled chicken wings, followed by gallic acid and ferulic acid [58]. However, for the DPPH scavenging activity, gallic acid was the most efficient, followed by protocatechuic acid and ferulic acid. Likewise, a total of eight phenolic compounds present in green tea extracts including epigallocatechin gallate, gallocatechin, catechin, epicatechin gallate, catechin gallate, eriodictyol, naringenin and quinic acid were found to inhibit the formation of eight PAHs (BaA, CHR, BbF, BkFL, BaP, DBahA, BghiP and IP) by 15–55% in charcoal-grilled chicken wings, with the phenolic compounds with weak antioxidant activity (quinic acid and naringenin) showing the highest inhibitory effects [21]. This outcome demonstrated that there is no direct relationship between free radical scavenging activity and PAH inhibition. In other words, some other factors may also involve in retarding PAH formation in meat products during heating.

Collectively, in this study we demonstrated that the addition of 0.5% cinnamon powder or 0.5% green tea powder was effective in inhibiting PAH formation in marinated pork through the antioxidant activity. However, no inhibition effect of PAH was observed in marinated juice, which may be due to degradation of phenolic compounds during heating to generate free radicals for the subsequent PAH formation [59]. Of the various PAHs formed in pork and juice during 24-h marinating, only DBahA and DBalP were classified as Group 2A [13]. This should pose no risk to human health as both PAHs were present in small amounts.

### 3.3. Principal Component Analysis

**Figure 5:**
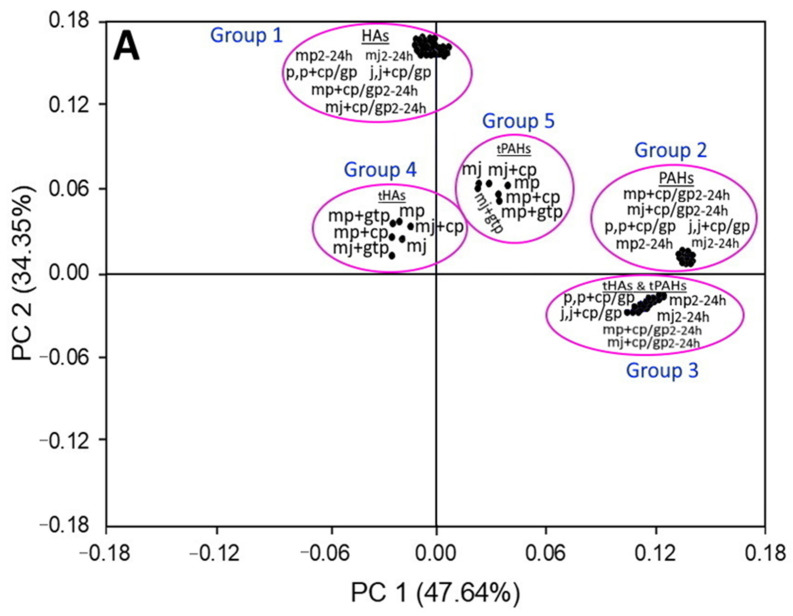

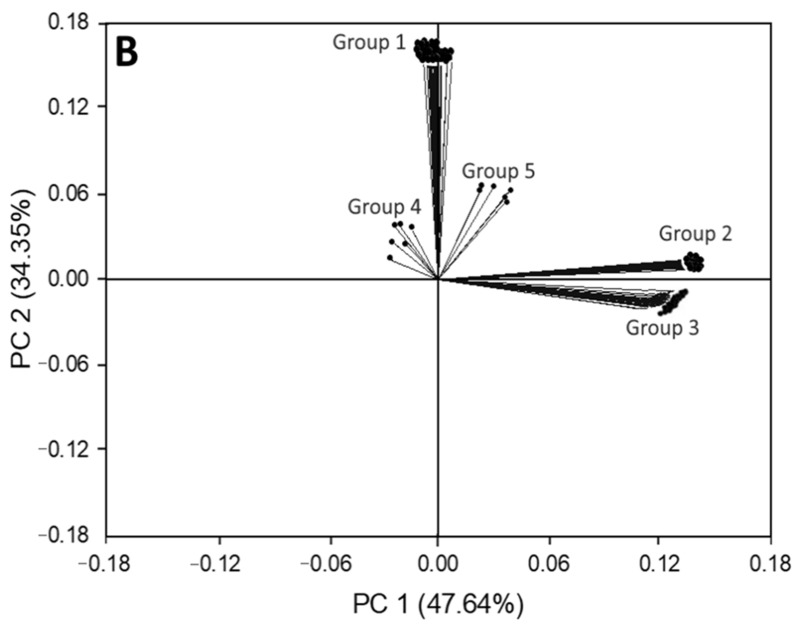
Principle component analysis showing score plot (**A**) and loading plot (**B**) for HAs and PAHs formation as affected by different flavorings and processing time length. Ground pork with different flavoring treatments at various marinating time length was performed by initially preparing the unmarinated juice by mixing 1% of sugar, 10% of soy sauce and 89% of deionized water (standard formula) without or with 0.5% cinnamon powder or 0.5% green tea powder, followed by adding 500 g of raw ground pork and marinating for 2, 4, 8, 12 and 24 h with the temperature controlled at 90 ± 2 °C. Mean values of triplicate determinations were used for PCA analysis. HAs, heterocyclic amines; tHAs, total HAs content; PAHs, polycyclic aromatic hydrocarbons; tPAHs, total PAHs content; p, unmarinated pork; j, unmarinated juice; p+cp/gp, unmarinated pork flavored with 0.5% cinnamon powder or 0.5% green tea powder; j+cp/gp, unmarinated juice flavored with 0.5% cinnamon powder or 0.5% green tea powder; mp2-24 h, marinated pork cooked at 90 ± 2 °C for different time length (2, 4, 8, 12 and 24 h); mj2-24 h, marinated juice cooked at 90 ± 2 °C for different time length (2, 4, 8, 12 and 24 h); mp+cp/gp2-24 h, marinated pork plus 0.5% cinnamon powder or 0.5% green tea powder cooked at 90 ± 2 °C for different time length (2, 4, 8, 12 and 24 h); mj+cp/gp2-24 h, marinated juice plus 0.5% cinnamon powder or 0.5% green tea powder cooked at 90 ± 2 °C for different time length (2, 4, 8, 12 and 24 h).

Figure 5 shows the PCA analysis of HAs and PAHs formation as affected by different flavorings (unmarinated pork/juice, marinated pork/juice, marinated pork/juice plus 0.5% cinnamon powder and marinated pork/juice plus 0.5% green tea powder) and marinating time length (2–24 h). Based on an eigen value >1, a total of four components (PCs 1–4) were shown to describe all the above treatments, with the first two components including PC 1 with 47.64% and PC 2 with 34.35% predominantly contributing to the maximum total variation of 81.99% in HAs or PAHs formation as affected by different flavorings and marinating time length. Analysis of score plot in Figure 5A revealed that the experimental data can be grouped into five groups with Groups 1 and 2 representing, respectively, various HAs and PAHs formed at 36 different treatments including unmarinated pork and juice (p, j), unmarinated pork and juice with 0.5% cinnamon powder/0.5% green tea powder (p+cp/gp, j+cp/gp), marinated pork and juice processed at five different time length (mp2-24 h, mj2-24 h), marinated pork and juice with 0.5% cinnamon powder/0.5% green tea powder at five different time length (mp+cp/gp2-24 h, mj+cp/gp2-24 h). Likewise, regardless of flavoring type, the total contents of HAs and PAHs formed at different marinating time length appeared together in serial line clusters in Group 3, confirming a time-dependent rise in the formation of both HAs and PAHs. On the other hand, for marinated pork/juice without and with 0.5% cinnamon powder/0.5% green tea powder, the total contents of HAs and PAHs formed were grouped as Group 4 and Group 5, respectively. All the five groups (Group 1–5) were well separated suggesting that both HAs and PAHs are formed by different mechanisms with their formation being significantly affected by various flavorings and marinating time length. Furthermore, the score plots in Group 1 were relatively more dispersed compared to the highly overlapping score plots in Group 2, affirming that HAs are more susceptible to formation than PAHs at different flavorings and marinating time length. 

To gain more insight on the correlation between the formation of HAs and PAHs, as affected by different flavorings and marinating time length, the loading plots in Figure 5B were analyzed based on inclination of Groups 1–5 towards PC 1 or PC 2 and the angle between them. The loading plots of formation of various PAHs at different flavoring and marinating time length (Group 2) and the contents of total HAs and total PAHs at different marinating time length (Group 3) as well as the contents of total PAHs at different flavorings (Group 5) were inclined to the same direction as PC 1 (47.64%); while that of various HAs formation at different flavorings and marinating time length (Group 1) and the contents of total HAs at different flavorings (Group 4) were inclined to the same direction as PC 2 (34.35%) implying their respective influence on PC 1 and PC 2. Moreover, the loading plots of various HAs and PAHs formed at different flavoring and marinating time length were diverged by a large degree of angle confirming again that their formation by different mechanism and a large variation in their contents. Likewise, comparatively the loading plots of total contents of HAs and PAHs as affected by marinating time length (Group 3) were diverged by a larger degree of angle from the loading plots of various HAs formed (Group 1) than that from various PAHs formed (Group 2), indicating that the formation of HAs is more favored than PAHs with increasing marinating time length. For different flavoring treatments, regardless of marinating time length, the loading plots of total contents of HAs and PAHs diverged by a reasonable angle to appear as two distinct groups (Group 4 and Group 5), revealing a significant variation in the impact of different flavorings on HAs and PAHs formation. Obviously, the incorporation of 0.5% cinnamon powder or 0.5% green tea powder was effective in inhibiting HAs formation, with the former showing a more pronounced inhibition effect for marinated pork and the latter showing a more efficient inhibition for marinated juice (Table 1). However, the addition of 0.5% cinnamon powder or 0.5% green tea powder was only effective in inhibiting PAH formation in marinated pork (Table 6). The aforementioned observations were well conformed with the discussion on HAs and PAHs formation as affected by different flavorings and marinating time length in Section 3.1 and Section 3.2 as well as the data tabulated in Table 1 and Table 6.

## 4. Conclusions

In conclusion, both HAs and PAHs were simultaneously extracted from pork by the QuEChERS method and subsequently analyzed UPLC-MS/MS and GC-MS/MS to study their formation and inhibition in ground pork and juice as affected by different flavorings and time length during marinating. Comparatively, HAs were more prone to formation than PAHs in marinated pork and juice. Following a rise in marinating time length, a time-dependent increase in HA and PAH contents in pork and juice were shown, which can be attributed to elevation in reducing sugar and creatinine levels for the former as well as benzaldehyde, 2-cyclohexene-1-one and trans,trans-2,4-decadienal levels for the latter during marinating. Addition of 0.5% cinnamon powder or 0.5% green tea powder effectively inhibited HA formation in both marinated pork and juice, whereas they were only effective in inhibiting PAH formation in marinated pork. Furthermore, the PCA analysis confirmed that both HAs and PAHs are formed by different mechanisms with their formation being significantly affected by different flavorings and marinating time length.

## Figures and Tables

**Table 1:** HAs contents (ng/g) in marinated pork and juice as affected different flavorings and time length.

HAs	0 h	2 h	4 h	8 h	12 h	24 h
Unmarinated pork/marinated pork ^1^						
DMIP	nd ^2^	nd	nd	0.21 ± 0.02 ^c^	0.367 ± 0.04 ^b^	0.86 ± 0.05 ^a^
Norharman	7.92 ± 0.30 ^f^	79.66 ± 2.27 ^e^	120.75 ± 5.37 ^d^	212.19 ± 9.04 ^c^	243.57 ± 9.41 ^b^	335.49 ± 15.04 ^a^
Phe-P-1	nd	23.98 ± 1.93 ^e^	30.57 ± 1.68 ^d^	64.66 ± 4.07 ^c^	118.57 ± 6.81 ^b^	189.12 ± 4.67 ^a^
Harman	53.51 ± 4.48 ^f^	403.18 ± 6.46 ^e^	686.35 ± 12.27 ^d^	1243.92 ± 11.53 ^c^	1624.68 ± 18.25 ^b^	2456.96 ± 41.45 ^a^
Glu-P-2	0.15 ± 0.01 ^c^	0.58 ± 0.09 ^b^	0.63 ± 0.10 ^b^	0.70 ± 0.08 ^b^	0.99 ± 0.10 ^a^	1.01 ± 0.16 ^a^
IQ [4,5-b]	nd	nd	nd	nd	0.15 ± 0.02 ^b^	1.53 ± 0.10 ^a^
IQx	nd	nd	0.21 ± 0.01 ^b^	0.24 ± 0.04 ^b^	0.28 ± 0.02 ^b^	0.47 ± 0.04 ^a^
8-MeIQx	nd	nd	nd	nd	trace ^3^	0.10 ± 0.02 ^a^
PhIP	nd	nd	0.24 ± 0.03 ^d^	0.32 ± 0.05 ^c^	0.68 ± 0.05 ^b^	0.95 ± 0.12 ^a^
Total	61.58 ± 4.69 ^f^	507.39 ± 10.69 ^e^	838.75 ± 15.97 ^d^	1522.23 ± 14.34 ^c^	1989.28 ± 33.09 ^b^	2986.47 ± 60.52 ^a^
Unmarinated pork/marinated pork + 0.5% cinnamon powder ^1^						
DMIP	nd	nd	0.20 ± 0.08 ^c^	0.37 ± 0.05 ^b^	0.42 ± 0.12 ^b^	0.98 ± 0.04 ^a^
Norharman	8.44 ± 0.61 ^f^	64.32 ± 3.47 ^e^	110.40 ± 8.48 ^d^	137.97 ± 6.25 ^c^	180.39 ± 3.75 ^b^	279.45 ± 8.89 ^a^
Phe-P-1	nd	4.27 ± 0.03 ^e^	9.87 ± 0.81 ^d^	17.78 ± 0.75 ^c^	31.31 ± 1.75 ^b^	69.67 ± 1.93 ^a^
Harman	50.18 ± 3.50 ^f^	285.69 ± 7.25 ^e^	607.12 ± 11.64 ^d^	891.23 ± 21.56 ^c^	1376.23 ± 26.68 ^b^	2287.77 ± 42.26 ^a^
Glu-P-2	0.20 ± 0.03 ^e^	0.50 ± 0.05 ^d^	0.65 ± 0.10 ^d^	0.87 ± 0.12 ^c^	1.47 ± 0.10 ^b^	1.73 ± 0.08 ^a^
IQ [4,5-b]	nd	nd	nd	nd	nd	1.62 ± 0.18 ^a^
IQx	nd	0.19 ± 0.01 ^c^	0.25 ± 0.03 ^c^	0.32 ± 0.05 ^b^	0.36 ± 0.05 ^b^	0.54 ± 0.02 ^a^
8-MeIQx	nd	nd	trace	trace	0.06 ± 0.01 ^b^	0.15 ± 0.04 ^a^
Total	58.82 ± 4.10 ^f^	354.98 ± 10.47 ^e^	728.48 ± 20.86 ^d^	1048.53 ± 28.13 ^c^	1590.24 ± 30.21 ^b^	2641.90 ± 44.61 ^a^
Unmarinated pork/marinated pork + 0.5% green tea powder ^1^						
DMIP	nd	nd	nd	0.25 ± 0.12 ^b^	0.34 ± 0.04 ^b^	0.50 ± 0.08 ^a^
Norharman	8.54 ± 0.53 ^e^	69.32 ± 3.60 ^d^	107.53 ± 5.06 ^c^	177.52 ± 6.34 ^b^	238.32 ± 19.22 ^a^	263.84 ± 28.33 ^a^
Phe-P-1	nd	16.35 ± 0.93 ^e^	29.40 ± 1.27 ^d^	61.43 ± 2.66 ^c^	88.39 ± 6.68 ^b^	174.72 ± 9.83 ^a^
Harman	52.03 ± 1.52 ^f^	346.31 ± 29.96 ^e^	629.19 ± 17.17 ^d^	1051.16 ± 29.38 ^c^	1606.89 ± 21.77 ^b^	2086.39 ± 24.34 ^a^
Glu-P-2	0.18 ± 0.06 ^c^	0.52 ± 0.15 ^b^	0.90 ± 0.07 ^a^	0.92 ± 0.09 ^a^	0.97 ± 0.15 ^a^	0.99 ± 0.18 ^a^
IQ [4,5-b]	nd	nd	nd	nd	nd	0.87 ± 0.10 ^a^
IQx	nd	nd	nd	0.25 ± 0.011 ^b^	0.28 ± 0.05 ^b^	0.45 ± 0.05 ^a^
8-MeIQx	nd	nd	nd	trace	trace	0.16 ± 0.03 ^a^
PhIP	nd	nd	nd	0.18 ± 0.02 ^c^	0.30 ± 0.09 ^b^	0.85 ± 0.05 ^a^
Total	60.76 ± 1.69 ^f^	432.50 ± 32.36 ^e^	767.02 ± 22.01 ^d^	1291.71 ± 34.05 ^c^	1935.50± 40.63 ^b^	2528.75 ± 57.76 ^a^
Unmarinated juice/marinated juice ^1^						
DMIP	nd ^2^	nd	nd	nd	0.27 ± 0.02 ^b^	1.33 ± 0.04 ^a^
Norharman	16.13 ± 0.68 ^f^	45.50 ± 0.626 ^e^	59.25 ± 2.26 ^d^	94.23 ± 3.06 ^c^	134.02 ± 5.93 ^b^	204.82 ± 8.24 ^a^
Phe-P-1	2.41 ± 0.17 ^d^	11.11 ± 0.49 ^c^	11.65 ± 0.85 ^c^	13.14 ± 0.55 ^c^	54.08 ± 2.70 ^b^	102.72 ± 4.67
Harman	120.42 ± 1.44 ^f^	304.32 ± 10.70 ^e^	373.96 ± 7.65 ^d^	598.07 ± 4.40 ^c^	1016.41 ± 9.09 ^b^	1481.22 ± 41.84 ^a^
Glu-P-2	0.30 ± 0.05 ^c^	0.35 ± 0.01 ^c^	0.57 ± 0.05 ^b^	0.59 ± 0.01 ^b^	0.59 ± 0.04 ^b^	0.96 ± 0.23 ^a^
IQ [4,5-b]	nd	nd	nd	nd	nd	trace ^3^
IQx	nd	0.18 ± 0.09 ^c^	0.26 ± 0.06 ^b^	0.26 ± 0.07 ^b^	0.36 ± 0.05 ^a^	0.37 ± 0.04 ^a^
8-MeIQx	nd	nd	nd	nd	0.06 ± 0.01 ^b^	0.18 ± 0.02 ^a^
PhIP	nd	nd	nd	0.17 ± 0.01 ^c^	0.22 ± 0.03 ^b^	0.48 ± 0.04 ^a^
Total	139.27 ± 2.16 ^f^	361.46 ± 11.02 ^e^	445.68 ± 10.17 ^d^	706.46 ± 4.68 ^c^	1206.01 ± 13.55 ^b^	1792.08 ± 54.65 ^a^
HAs (ng/g)	Unmarinated juice/marinated juice + 0.5% cinnamon powder ^1^					
DMIP	nd	nd	nd	nd	nd	0.51 ± 0.03 ^a^
Norharman	16.91 ± 0.38 ^e^	45.54 ± 2.20 ^d^	48.39 ± 2.70	77.62 ± 5.76 ^c^	116.80 ± 3.50 ^b^	141.38 ± 5.40 ^a^
Phe-P-1	1.72 ± 0.14 ^e^	9.95 ± 0.19 ^d^	18.95 ± 0.93 ^c^	19.53 ± 1.22 ^c^	23.64 ± 1.53 ^b^	73.03 ± 3.93 ^a^
Harman	116.23 ± 3.35 ^f^	273.02 ± 6.80 ^e^	309.93 ± 5.27 ^d^	538.04 ± 7.62 ^c^	900.94 ± 14.20 ^b^	1065.64 ± 22.30 ^a^
Glu-P-2	0.38 ± 0.06 ^c^	0.36 ± 0.01 ^c^	0.53 ± 0.08 ^b^	0.55 ± 0.05 ^b^	0.61 ± 0.03 ^b^	0.71 ± 0.04 ^a^
IQ [4,5-b]	nd	nd	nd	nd	nd	0.22 ± 0.04 ^a^
IQx	nd	0.21 ± 0.01 ^c^	0.22 ± 0.06 ^c^	0.36 ± 0.04 ^b^	0.34 ± 0.03 ^b^	0.42 ± 0.06 ^a^
8-MeIQx	nd	nd	nd	trace	trace	0.10 ± 0.03 ^a^
PhIP	nd	nd	nd	nd	0.13 ± 0.02 ^b^	0.32 ± 0.02 ^a^
Total	135.23 ± 3.47 ^f^	329.08 ± 9.15 ^e^	378.02 ± 7.84 ^d^	636.10 ± 11.33 ^c^	1042.46 ± 19.24 ^b^	1282.32 ± 30.81 ^a^
HAs (ng/g)	Unmarinated juice/marinated juice + 0.5% green tea powder ^1^					
DMIP	nd	nd	nd	nd	nd	0.98 ± 0.05 ^a^
Norharman	18.23 ± 0.88 ^e^	23.21 ± 2.05 ^e^	32.39 ± 2.26 ^d^	50.15 ± 2.89 ^c^	92.06 ± 3.47 ^b^	130.70 ± 5.45 ^a^
Phe-P-1	0.32 ± 0.07 ^d^	0.50 ± 0.04 ^d^	0.91 ± 0.17 ^cd^	1.63 ± 0.26 ^c^	7.47 ± 0.41 ^b^	15.83 ± 1.41 ^a^
Harman	118.63 ± 2.33 ^f^	137.13 ± 7.52 ^e^	202.93 ± 4.54 ^d^	385.25 ± 25.68 ^c^	778.41 ± 35.98 ^b^	1249.16 ± 31.11 ^a^
Glu-P-2	0.35 ± 0.05 ^d^	0.47 ± 0.04 ^cd^	0.56 ± 0.06 ^c^	0.70 ± 0.01 ^b^	0.70 ± 0.09 ^b^	0.96 ± 0.13 ^a^
IQ [4,5-b]	nd	nd	nd	nd	nd	0.45 ± 0.05 ^a^
IQx	nd	nd	0.21 ± 0.03 ^b^	0.27 ± 0.02 ^b^	0.27 ± 0.06 ^b^	0.37 ± 0.07 ^a^
8-MeIQx	nd	nd	nd	trace	trace	0.10 ± 0.02 ^a^
Total	137.52 ± 3.24 ^e^	161.30 ± 9.24 ^e^	237.001 ± 6.431 ^d^	438.00 ± 28.570 ^c^	878.91 ± 39.72 ^b^	1398.56 ± 38.23 ^a^

^1^ Data are presented as mean ± standard deviation of triplicate determinations and data with different small letters (a–f) in the same row are significantly different (*p* < 0.05); ^2^ not detected; ^3^ LOQ ≥ PAHs levels ≥ LOD.

**Table 2:** Individual and total amino acid (AA) contents in unmarinated/marinated pork and juice as affected by different flavorings and time length.

AA (mg/g)	0 h	2 h	4 h	8 h	12 h	24 h
marinated pork ^1^						
Asp	0.41 ± 0.04	0.40 ± 0.59	0.36 ± 0.01	0.40 ± 0.01	0.28 ± 0.02	0.41 ± 0.02
Glu	0.94 ± 0.05	0.77 ± 0.00	0.76 ± 0.04	0.82 ± 0.03	0.69 ± 0.01	0.77 ± 0.01
Ser	0.23 ± 0.01	0.21 ± 0.00	0.20 ± 0.00	0.21 ± 0.00	0.19 ± 0.00	0.21 ± 0.00
His	0.06 ± 0.01	0.05 ± 0.01	0.04 ± 0.01	0.05 ± 0.00	0.08 ± 0.01	0.11 ± 0.00
Gly	0.50 ± 0.02	0.46 ± 0.03	0.48 ± 0.04	0.38 ± 0.01	0.46 ± 0.05	0.43 ± 0.01
Thr	0.09 ± 0.01	0.09 ± 0.00	0.08 ± 0.01	0.10 ± 0.00	0.14 ± 0.01	0.16 ± 0.02
Arg	0.49 ± 0.01	0.42 ± 0.01	0.42 ± 0.02	0.40 ± 0.01	0.42 ± 0.01	0.42 ± 0.00
Ala	0.47 ± 0.01	0.43 ± 0.00	0.43 ± 0.03	0.41 ± 0.01	0.39 ± 0.00	0.42 ± 0.00
Tyr	0.22 ± 0.00 ^2^	0.06 ± 0.01	0.05 ± 0.02	0.05 ± 0.01	0.30 ± 0.02	0.16 ± 0.00
Cys	0.15 ± 0.01	0.11 ± 0.00	0.11 ± 0.00	0.11 ± 0.00	0.03 ± 0.00	0.05 ± 0.00
Val	0.23 ± 0.04	0.24 ± 0.00	0.20 ± 0.06	0.28 ± 0.04	0.17 ± 0.01	0.19 ± 0.02
Met	0.23 ± 0.14	0.14 ± 0.00	0.15 ± 0.01	0.23 ± 0.07	0.14 ± 0.00	0.16 ± 0.01
Phe	0.21 ± 0.01	0.23 ± 0.00	0.23 ± 0.01	0.20 ± 0.03	0.17 ± 0.01	0.20 ± 0.00
Ile	0.24 ± 0.06	0.28 ± 0.00	0.27 ± 0.01	0.23 ± 0.06	0.30 ± 0.01	0.27 ± 0.01
Leu	0.54 ± 0.20	0.66 ± 0.01	0.69 ± 0.03	0.67 ± 0.02	0.50 ± 0.01	0.55 ± 0.01
Lys	0.53 ± 0.05	0.55 ± 0.01	0.59 ± 0.04	0.61 ± 0.02	0.55 ± 0.04	0.53 ± 0.02
Pro	0.26 ± 0.00	0.40 ± 0.02	0.41 ± 0.04	0.45 ± 0.01	0.43 ± 0.03	0.42 ± 0.01
Total	5.81 ± 0.14	5.83 ± 0.63	5.45 ± 0.10	5.61 ± 0.03	5.24± 0.02	5.45 ± 0.04
marinated pork + 0.5% cinnamon powder ^1^						
Asp	0.42 ± 0.01	0.40 ± 0.02	0.36 ± 0.01	0.38 ± 0.01	0.41 ± 0.01	0.36 ± 0.01
Glu	0.78 ± 0.02	0.72 ± 0.02	0.68 ± 0.00	0.74 ± 0.00	0.78 ± 0.00	0.80 ± 0.01
Ser	0.21 ± 0.00	0.22 ± 0.00	0.21 ± 0.00	0.20 ± 0.00	0.22 ± 0.00	0.21 ± 0.00
His	0.03 ± 0.01	0.08 ± 0.00	0.06 ± 0.00	0.09 ± 0.00	0.04 ± 0.00	0.04 ± 0.00
Gly	0.58 ± 0.02	0.55 ± 0.04	0.13 ± 0.01	0.13 ± 0.01	0.14 ± 0.01	0.10 ± 0.00
Thr	0.14 ± 0.01	0.15 ± 0.00	0.42 ± 0.01	0.43 ± 0.00	0.44 ± 0.00	0.49 ± 0.00
Arg	0.53 ± 0.00	0.48 ± 0.00	0.96 ± 0.00	0.98 ± 0.01	1.02 ± 0.01	1.18 ± 0.00
Ala	0.44 ± 0.00	0.44 ± 0.00	0.40 ± 0.00	0.41 ± 0.00	0.43 ± 0.01	0.49 ± 0.00
Tyr	0.13 ± 0.00	0.14 ± 0.00	0.13 ± 0.00	0.15 ± 0.00	0.16 ± 0.00	0.14 ± 0.00
Cys	0.14 ± 0.01	0.14 ± 0.00	0.04 ± 0.01	0.06 ± 0.00	0.06 ± 0.00	0.04 ± 0.00
Val	0.18 ± 0.00	0.16 ± 0.01	0.16 ± 0.00	0.18 ± 0.00	0.20 ± 0.00	0.17 ± 0.01
Met	0.16 ± 0.01	0.15 ± 0.00	0.14 ± 0.00	0.15 ± 0.00	0.16 ± 0.00	0.13 ± 0.00
Phe	0.23 ± 0.07	0.19 ± 0.01	0.17 ± 0.00	0.19 ± 0.00	0.21 ± 0.00	0.21 ± 0.01
Ile	0.36 ± 0.16	0.25 ± 0.01	0.28 ± 0.01	0.25 ± 0.01	0.27 ± 0.01	0.27 ± 0.01
Leu	0.43 ± 0.09	0.56 ± 0.00	0.49 ± 0.01	0.53 ± 0.01	0.56 ± 0.02	0.65 ± 0.01
Lys	0.45 ± 0.06	0.58 ± 0.04	0.60 ± 0.01	0.56 ± 0.01	0.55 ± 0.01	0.44 ± 0.01
Pro	0.29 ± 0.02	0.56 ± 0.00	0.44 ± 0.01	0.43 ± 0.01	0.43 ± 0.01	0.38 ± 0.00
Total	5.49 ± 0.11	5.79 ± 0.10	5.56 ± 0.02	5.85 ± 0.06	6.07 ± 0.04	6.10 ± 0.07
marinated pork + 0.5% green tea powder ^1^						
Asp	0.53 ± 0.05	0.43 ± 0.09	0.41 ± 0.08	0.65 ± 0.37	0.36 ± 0.06	0.34 ± 0.08
Glu	0.94 ± 0.08	0.81 ± 0.21	0.99 ± 0.23	1.04 ± 0.27	1.04 ± 0.22	1.06 ± 0.26
Ser	0.23 ± 0.02	0.24 ± 0.05	0.23 ± 0.05	0.23 ± 0.05	0.24 ± 0.05	0.23 ± 0.06
His	0.04 ± 0.00	0.04 ± 0.00	0.03 ± 0.01	0.04 ± 0.00	0.03 ± 0.01	0.03 ± 0.01
Gly	0.12 ± 0.01	0.14 ± 0.02	0.18 ± 0.06	0.17 ± 0.06	0.19 ± 0.07	0.17 ± 0.07
Thr	0.44 ± 0.01	0.46 ± 0.08	0.53 ± 0.08	0.48 ± 0.08	0.57 ± 0.08	0.56 ± 0.08
Arg	1.13 ± 0.06	1.10 ± 0.20	1.19 ± 0.23	1.24 ± 0.25	1.33 ± 0.23	1.32 ± 0.23
Ala	0.48 ± 0.03	0.46 ± 0.09	0.50 ± 0.10	0.52 ± 0.11	0.56 ± 0.10	0.56 ± 0.10
Tyr	0.01 ± 0.00	0.01 ± 0.00	0.14 ± 0.03	0.01 ± 0.00	0.09 ± 0.02	0.20 ± 0.04
Cys	0.01 ± 0.00	0.07 ± 0.04	0.08 ± 0.00	0.06 ± 0.00	0.09 ± 0.01	0.08 ± 0.01
Val	0.23 ± 0.02	0.19 ± 0.03	0.12 ± 0.04	0.24 ± 0.05	0.24 ± 0.05	0.22 ± 0.06
Met	0.14 ± 0.02	0.17 ± 0.03	0.21 ± 0.07	0.17 ± 0.05	0.22 ± 0.06	0.22 ± 0.07
Phe	0.16 ± 0.01	0.18 ± 0.03	0.24 ± 0.05	0.20 ± 0.03	0.24 ± 0.03	0.24 ± 0.04
Ile	0.36 ± 0.04	0.34 ± 0.09	0.23 ± 0.01	0.38 ± 0.11	0.43 ± 0.13	0.36 ± 0.09
Leu	0.52 ± 0.03	0.57 ± 0.11	0.45 ± 0.08	0.60 ± 0.19	0.70 ± 0.13	0.72 ± 0.13
Lys	0.50 ± 0.15	0.71 ± 0.20	0.71 ± 0.18	0.57 ± 0.14	0.64 ± 0.18	0.57 ± 0.13
Pro	0.31 ± 0.01	0.42 ± 0.04	0.38 ± 0.02	0.27 ± 0.02	0.35 ± 0.02	0.35 ± 0.01
Total	6.16 ± 0.51	6.36 ± 1.14	6.54 ± 1.00	6.63 ± 1.41	7.25 ± 1.32	7.23 ± 1.42
marinated juice ^1^						
Asp	0.41 ± 0.04	0.71 ± 0.59	0.36 ± 0.01	0.40 ± 0.01	0.28 ± 0.02	0.41 ± 0.02
Glu	0.94 ± 0.05	0.77 ± 0.00	0.76 ± 0.04	0.82 ± 0.03	0.69 ± 0.01	0.77 ± 0.01
Ser	0.23 ± 0.01	0.21 ± 0.00	0.20 ± 0.00	0.21 ± 0.00	0.19 ± 0.00	0.21 ± 0.00
His	0.45 ± 0.27	0.79 ± 0.50	0.83 ± 0.54	0.73 ± 0.50	0.71 ± 0.49	0.69 ± 0.45
Gly	0.50 ± 0.02	0.46 ± 0.03	0.48 ± 0.04	0.38 ± 0.01	0.46 ± 0.05	0.43 ± 0.01
Thr	0.09 ± 0.01	0.09 ± 0.00	0.08 ± 0.01	0.10 ± 0.00	0.14 ± 0.01	0.16 ± 0.02
Arg	0.49 ± 0.01	0.42 ± 0.01	0.42 ± 0.02	0.40 ± 0.01	0.42 ± 0.01	0.42 ± 0.00
Ala	0.47 ± 0.01	0.43 ± 0.00	0.43 ± 0.03	0.41 ± 0.01	0.39 ± 0.00	0.42 ± 0.00
Tyr	0.22 ± 0.00	0.06 ± 0.01	0.05 ± 0.02	0.05 ± 0.01	0.30 ± 0.02	0.16 ± 0.00
Cys	0.15 ± 0.01	0.11 ± 0.00	0.11 ± 0.00	0.11 ± 0.00	0.03 ± 0.00	0.05 ± 0.00
Val	0.23 ± 0.04	0.24 ± 0.00	0.20 ± 0.06	0.28 ± 0.04	0.17 ± 0.01	0.19 ± 0.02
Met	0.23 ± 0.14	0.14 ± 0.00	0.15 ± 0.01	0.23 ± 0.07	0.14 ± 0.00	0.16 ± 0.01
Phe	0.21 ± 0.01	0.23 ± 0.00	0.23 ± 0.01	0.20 ± 0.03	0.17 ± 0.01	0.20 ± 0.00
Ile	0.24 ± 0.06	0.28 ± 0.00	0.27 ± 0.01	0.23 ± 0.06	0.30 ± 0.01	0.27 ± 0.01
Leu	0.54 ± 0.20	0.66 ± 0.01	0.69 ± 0.03	0.67 ± 0.02	0.50 ± 0.01	0.55 ± 0.01
Lys	0.53 ± 0.05	0.55 ± 0.01	0.59 ± 0.04	0.61 ± 0.02	0.55 ± 0.04	0.53 ± 0.02
Pro	0.26 ± 0.00	0.40 ± 0.02	0.41 ± 0.04	0.45 ± 0.01	0.43 ± 0.03	0.42 ± 0.01
Total	12.44 ± 1.64	13.11 ± 2.06	14.26 ± 3.04	14.94 ± 3.61	14.17 ± 3.05	13.86 ± 2.95
marinated juice + 0.5% cinnamon powder ^1^						
Asp	0.42 ± 0.01	0.40 ± 0.02	0.36 ± 0.01	0.38 ± 0.01	0.41 ± 0.01	0.36 ± 0.01
Glu	0.78 ± 0.02	0.72 ± 0.02	0.68 ± 0.00	0.74 ± 0.00	0.78 ± 0.00	0.80 ± 0.01
Ser	0.21 ± 0.00	0.22 ± 0.00	0.21 ± 0.00	0.20 ± 0.00	0.22 ± 0.00	0.21 ± 0.00
His	0.55 ± 0.36	0.70 ± 0.45	0.71 ± 0.52	0.62 ± 0.46	0.58 ± 0.38	0.53 ± 0.34
Gly	0.58 ± 0.02	0.55 ± 0.04	0.13 ± 0.01	0.13 ± 0.01	0.14 ± 0.01	0.10 ± 0.00
Thr	0.14 ± 0.01	0.15 ± 0.00	0.42 ± 0.01	0.43 ± 0.00	0.44 ± 0.00	0.49 ± 0.00
Arg	0.53 ± 0.00	0.48 ± 0.00	0.96 ± 0.00	0.98 ± 0.01	1.02 ± 0.02	1.18 ± 0.00
Ala	0.44 ± 0.00	0.44 ± 0.00	0.40 ± 0.00	0.41 ± 0.00	0.43 ± 0.01	0.49 ± 0.00
Tyr	0.13 ± 0.00	0.14 ± 0.00	0.07 ± 0.02	0.09 ± 0.03	0.09 ± 0.03	0.10 ± 0.03
Cys	0.14 ± 0.01	0.14 ± 0.00	0.16 ± 0.13	0.18 ± 0.16	0.16 ± 0.01	0.12 ± 0.04
Val	0.18 ± 0.00	0.16 ± 0.01	0.16 ± 0.00	0.18 ± 0.00	0.20 ± 0.00	0.17 ± 0.01
Met	0.16 ± 0.01	0.15 ± 0.00	0.14 ± 0.00	0.15 ± 0.00	0.16 ± 0.00	0.13 ± 0.00
Phe	0.23 ± 0.07	0.19 ± 0.01	0.17 ± 0.00	0.19 ± 0.00	0.21 ± 0.00	0.21 ± 0.01
Ile	0.36 ± 0.16	0.25 ± 0.01	0.28 ± 0.01	0.25 ± 0.01	0.27 ± 0.01	0.27 ± 0.01
Leu	0.43 ± 0.09	0.56 ± 0.00	0.49 ± 0.01	0.53 ± 0.01	0.56 ± 0.02	0.65 ± 0.01
Lys	0.45 ± 0.06	0.58 ± 0.04	0.60 ± 0.01	0.56 ± 0.01	0.55 ± 0.01	0.44 ± 0.01
Pro	0.29 ± 0.02	0.56 ± 0.00	0.44 ± 0.01	0.43 ± 0.01	0.43 ± 0.01	0.38 ± 0.00
Total	13.47 ± 2.56	13.61 ± 1.25	14.41 ± 3.46	14.85 ± 4.18	15.19 ± 4.37	15.25 ± 3.98
marinated juice + 0.5% green tea powder ^1^						
Asp	0.53 ± 0.05	0.43 ± 0.09	0.41 ± 0.08	0.65 ± 0.37	0.36 ± 0.06	0.34 ± 0.08
Glu	0.94 ± 0.08	0.81 ± 0.21	0.99 ± 0.23	1.04 ± 0.27	1.04 ± 0.22	1.06 ± 0.26
Ser	0.23 ± 0.02	0.24 ± 0.05	0.23 ± 0.05	0.23 ± 0.05	0.24 ± 0.05	0.23 ± 0.06
His	0.37 ± 0.20	0.49 ± 0.30	0.44 ± 0.31	0.30 ± 0.18	0.30 ± 0.19	0.28 ± 0.15
Gly	0.12 ± 0.01	0.14 ± 0.02	0.18 ± 0.06	0.17 ± 0.06	0.19 ± 0.07	0.17 ± 0.07
Thr	0.44 ± 0.01	0.46 ± 0.08	0.53 ± 0.08	0.48 ± 0.08	0.57 ± 0.08	0.56 ± 0.08
Arg	1.13 ± 0.06	1.10 ± 0.20	1.19 ± 0.23	1.24 ± 0.25	1.33 ± 0.23	1.32 ± 0.23
Ala	0.48 ± 0.03	0.46 ± 0.09	0.50 ± 0.10	0.52 ± 0.11	0.56 ± 0.10	0.56 ± 0.10
Tyr	0.15 ± 0.01	0.07 ± 0.02	0.09 ± 0.03	0.12 ± 0.05	0.09 ± 0.02	0.20 ± 0.04
Cys	0.14 ± 0.03	0.36 ± 0.23	0.24 ± 0.09	0.05 ± 0.01	0.09 ± 0.01	0.08 ± 0.01
Val	0.23 ± 0.02	0.19 ± 0.03	0.12 ± 0.04	0.24 ± 0.05	0.24 ± 0.05	0.22 ± 0.06
Met	0.14 ± 0.02	0.17 ± 0.03	0.21 ± 0.07	0.17 ± 0.05	0.22 ± 0.06	0.22 ± 0.07
Phe	0.16 ± 0.01	0.18 ± 0.03	0.24 ± 0.05	0.20 ± 0.03	0.24 ± 0.03	0.24 ± 0.04
Ile	0.36 ± 0.04	0.34 ± 0.09	0.23 ± 0.01	0.38 ± 0.11	0.43 ± 0.13	0.36 ± 0.09
Leu	0.52 ± 0.03	0.57 ± 0.11	0.45 ± 0.08	0.60 ± 0.19	0.70 ± 0.13	0.72 ± 0.13
Lys	0.50 ± 0.15	0.71 ± 0.20	0.71 ± 0.18	0.57 ± 0.14	0.64 ± 0.18	0.57 ± 0.13
Pro	0.31 ± 0.01	0.42 ± 0.04	0.38 ± 0.02	0.27 ± 0.02	0.35 ± 0.02	0.35 ± 0.01
Total	11.88 ± 0.95	13.66 ± 2.60	14.93 ± 3.97	15.58 ± 2.23	16.38 ± 5.75	16.65 ± 5.50

^1^ Data are presented as mean ± standard deviation of triplicate determinations. ^2^ Data with the standard deviation values <0.005 are shown as ±0.00 to maintain 2 significant digits.

**Table 3:** Changes of HA precursor contents in unmarinated/marinated pork and juice as affected by different flavorings and time length.

Time Length	0 h	2 h	4 h	8 h	12 h	24 h
Reducing sugar content (mg/g) ^1^						
marinated pork (MP)	0.09 ± 0.25 ^e,A^	1.37 ± 0.46 ^d,B^	1.12 ± 0.08 ^d,B^	2.32 ± 0.28 ^c,B^	3.50 ± 0.02 ^b,C^	4.03 ± 0.05 ^a,C^
MP + 0.5% cinnamon powder	0.08 ± 0.48 ^c,A^	1.56 ± 1.70 ^b,A^	1.03 ± 0.44 ^b,B^	1.83 ± 0.37 ^b,C^	4.73 ± 0.10 ^a,B^	5.33 ± 0.66 ^a,B^
MP + 0.5% green tea powder	0.07 ± 0.40 ^d,A^	1.22 ± 0.76 ^c,B^	4.08 ± 1.65 ^b,A^	3.75 ± 0.47 ^b,A^	6.73 ± 1.81 ^a,A^	6.50 ± 0.21 ^a,A^
Creatine content (mg/100 g) ^1^						
marinated pork (MP)	57.85 ± 0.21 ^a,A^	25.70 ± 0.1 ^b,A^	20.43 ± 0.14 ^c,A^	15.94 ± 0.20 ^d,A^	11.34 ± 0.08 ^e,A^	6.72 ± 0.07 ^f,C^
MP + 0.5% cinnamon powder	56.99 ± 0.61 ^a,A^	22.98 ± 0.13 ^b,B^	14.12 ± 0.10 ^c,B^	11.82 ± 0.17 ^d,B^	8.96 ± 0.08 ^e,B^	7.27 ± 0.08 ^f,B^
MP + 0.5% green tea powder	57.14 ± 0.37 ^a,A^	21.16 ± 0.12 ^b,C^	13.46 ± 0.56 ^c,B^	10.66 ± 0.08 ^d,C^	8.72 ± 0.09 ^e,C^	7.62 ± 0.06 ^f,A^
Creatinine content (mg/100 g) ^1^						
marinated pork (MP)	5.97 ± 0.15 ^e,A^	34.57 ± 0.54 ^d,B^	60.33 ± 1.23 ^b,A^	76.43 ± 2.74 ^a,A^	60.26 ± 1.55 ^b,A^	44.47 ± 0.92 ^c,B^
MP + 0.5% cinnamon powder	5.84 ± 0.29 ^e,A^	37.07 ± 0.06 ^d,A^	46.38 ± 0.17 ^c,C^	56.60 ± 0.64 ^a,B^	50.22 ± 0.26 ^b,C^	45.43 ± 1.72 ^c,B^
MP + 0.5% green tea powder	5.11 ± 0.47 ^d,A^	37.17 ± 0.26 ^c,A^	49.03 ± 1.12 ^b,B^	51.65 ± 0.47 ^a,C^	51.57 ± 0.14 ^a,B^	49.31 ± 1.25 ^b,A^
Reducing sugar content (mg/g) ^1^						
marinated juice (MJ)	0.15 ± 0.06 ^d,A^	12.18 ± 0.13 ^a,A^	9.30 ± 0.22 ^b,B^	9.95 ± 0.29 ^b,B^	7.74 ± 0.17 ^c,C^	7.89 ± 0.11 ^c,C^
MJ + 0.5% cinnamon powder	0.10 ± 0.04 ^e,A^	11.01 ± 0.07 ^b,B^	10.91 ± 0.14 ^b,A^	11.61 ± 0.19 ^a,A^	9.43 ± 0.24 ^d,A^	9.74 ± 0.17 ^c,A^
MJ + 0.5% green tea powder	0.13 ± 0.02 ^e,A^	8.92 ± 0.06 ^a,C^	7.96 ± 0.08 ^d,C^	7.87 ± 0.09 ^d,C^	8.76 ± 0.10 ^b,B^	8.57 ± 0.09 ^c,B^
Creatine content (mg/100 g) ^1^						
marinated juice (MJ)	23.30 ± 0.18 ^a,A^	22.95 ± 0.07 ^b,A^	22.28 ± 0.06 ^c,A^	16.57 ± 0.24 ^d,A^	10.42 ± 0.15 ^e,B^	9.30 ± 0.14 ^f,,B^
MJ + 0.5% cinnamon powder	23.21 ± 0.03 ^a,A^	22.46 ± 0.02 ^b,B^	18.18 ± 0.08 ^c,B^	14.44 ± 0.49 ^d,B^	13.38 ± 0.13 ^e,A^	10.26 ± 0.02 ^f,A^
MJ + 0.5% green tea powder	23.01 ± 0.21 ^a,A^	20.43 ± 1.22 ^b,C^	15.84 ± 0.38 ^c,C^	13.14 ± 0.15 ^d,C^	10.21 ± 0.10 ^e,B^	10.79 ± 0.72 ^e,A^
Creatinine content (mg/100 g) ^1^						
marinated juice (MJ)	nd ^2^	6.47 ± 0.10 ^e,B^	11.23 ± 0.23 ^b,A^	14.44 ± 0.52 ^a,A^	10.07 ± 0.26 ^c,A^	8.20 ± 0.17 ^d,B^
MJ + 0.5% cinnamon powder	nd	6.77 ± 0.01 ^d,A^	8.51 ± 0.03 ^c,C^	10.74 ± 0.12 ^a,B^	9.17 ± 0.05 ^b,C^	8.29 ± 0.31 ^c,B^
MJ + 0.5% green tea powder	nd	6.83 ± 0.05 ^d,A^	9.17 ± 0.21 ^c,B^	9.76 ± 0.09 ^a,C^	9.51 ± 0.03 ^ab,B^	9.37 ± 0.23 ^bc,A^

^1^ Data are presented as mean ± standard deviation of triplicate determinations and data with different small letters (a–f) in the same row and capital letters (A–C) in the same column are significantly different (*p* < 0.05). ^2^ not detected.

**Table 4:** Antioxidant capacity as well as Maillard browning index of marinated pork and juice as affected by different flavorings and time length.

Time Length	0 h	2 h	4 h	8 h	12 h	24 h
FRAP (TAC, mmol Trolox equivalent/kg) ^1^						
marinated pork (MP)	11.75 ± 0.17 ^f,B^	13.84 ± 0.21 ^e,C^	18.98 ± 0.23 ^d,C^	24.77 ± 0.14 ^c,C^	25.51 ± 0.20 ^b,C^	28.90 ± 0.70 ^a,C^
MP + 0.5% cinnamon powder	11.96 ± 0.19 ^f,B^	20.13 ± 0.20 ^e,B^	27.32 ± 0.64 ^d,B^	35.71 ± 0.69 ^c,B^	37.22 ± 0.49 ^b,B^	42.74 ± 0.34 ^a,B^
MP + 0.5% green tea powder	21.33 ± 0.29 ^f,A^	49.97 ± 0.52 ^c,A^	43.24 ± 0.77 ^e,A^	54.27 ± 0.17 ^a,A^	51.08 ± 0.05 ^b,A^	47.09 ± 0.58 ^d,A^
DPPH (TAC, mmol Trolox equivalent/kg) ^1^						
marinated pork (MP)	28.94 ± 0.11 ^c,C^	27.10 ± 0.22 ^e,C^	27.37 ± 0.13 ^d,B^	30.13 ± 0.07 ^b,C^	30.28 ± 0.08 ^b,B^	30.63 ± 0.09 ^a,B^
MP + 0.5% cinnamon powder	29.26 ± 0.19 ^d,B^	29.47 ± 0.07 ^c,B^	30.85 ± 0.02 ^ab,A^	30.78 ± 0.02 ^b,B^	30.83 ± 0.09 ^ab,A^	30.97 ± 0.09 ^a,A^
MP + 0.5% green tea powder	30.58 ± 0.07 ^c,A^	31.02 ± 0.04 ^a,A^	30.84 ± 0.06 ^ab,A^	31.01 ± 0.05 ^a,A^	30.75 ± 0.33 ^bc,A^	30.93 ± 0.02 ^ab,A^
FRAP (TAC, mmol Trolox equivalent/kg) ^1^						
marinated juice (MJ)	54.33 ± 0.96 ^a,C^	49.30 ± 0.47 ^c,C^	50.76 ± 0.19 ^b,C^	54.87 ± 0.64 ^a,C^	44.04 ± 0.91 ^e,C^	45.99 ± 1.09 ^d,C^
MJ + 0.5% cinnamon powder	56.27 ± 1.11 ^b,B^	52.87 ± 0.83 ^c,B^	53.50 ± 0.37 ^c,B^	57.62 ± 0.32 ^a,B^	58.31 ± 0.36 ^a,B^	51.36 ± 0.51 ^d,B^
MJ + 0.5% green tea powder	67.13 ± 0.14 ^b,A^	69.64 ± 0.15 ^a,A^	69.37 ± 0.46 ^a,A^	66.18 ± 0.22 ^c,A^	62.98 ± 0.81 ^d,A^	62.98 ± 0.81 ^d,A^
DPPH (TAC, mmol Trolox equivalent/kg) ^1^						
marinated juice (MJ)	30.32 ± 0.03 ^a,A^	29.94 ± 0.52 ^ab,A^	30.13 ± 0.03 ^a,A^	30.06 ± 0.05 ^a,A^	30.12 ± 0.11 ^a,A^	29.66 ± 0.07 ^b,A^
MJ + 0.5% cinnamon powder	30.41 ± 0.02 ^a,A^	30.23 ± 0.18 ^ab,A^	30.02 ± 0.25 ^b,A^	30.02 ± 0.05 ^b,A^	29.71 ± 0.06 ^c,B^	29.65 ± 0.02 ^c,A^
MJ + 0.5% green tea powder	30.18 ± 0.05 ^b,A^	30.34 ± 0.09 ^a,A^	30.11 ± 0.02 ^b,A^	29.95 ± 0.09 ^c,A^	29.68 ± 0.02 ^d,B^	29.50 ± 0.03 ^e,A^
Maillard browning index ^1^	0 h	2 h	4 h	8 h	12 h	24 h
marinated juice (MJ)	0.21 ± 0.01 ^d,B^	0.26 ± 0.00 ^c,A,2^	0.30 ± 0.01 ^b,A^	0.31 ± 0.01 ^b,A^	0.31 ± 0.01 ^b,B^	0.38 ± 0.01 ^a,B^
MJ + 0.5% cinnamon powder	0.23 ± 0.01 ^d,A^	0.26 ± 0.02 ^d,A^	0.26 ± 0.03 ^d,A^	0.33 ± 0.01 ^c,A^	0.38 ± 0.01 ^b,B^	0.49 ± 0.02 ^a,AB^
MJ + 0.5% green tea powder	0.26 ± 0.01 ^b,A^	0.25 ± 0.02 ^b,A^	0.26 ± 0.05 ^b,A^	0.35 ± 0.07 ^b,A^	0.53 ± 0.07 ^a,A^	0.56 ± 0.11 ^a,A^

^1^ Data are presented as mean ± standard deviation of triplicate determinations and data with different small letters (a–f) in the same row and capital letters (A–C) in the same column are significantly different (*p* < 0.05). ^2^ Data with the standard deviation values <0.005 are shown as ±0.00 to maintain 2 significant digits.

**Table 5:** Analysis of the bioactive compounds (mg/g) in curcuma powder and cinnamon powder.

**Cinnamon Powder**										
**Compound**	**Neochlorogenic Acid**	**Benzoic Acid**	**Caffeic Acid**	**Hyperoside + Isoquecetin**	**Coumarin**	**Quercetin**	**Cinnamic** **Acid**	**Cinnam-Aldehyde**	**Eugenol**	**p-Coumaric Acid**
Content (mg/g) ^1^	0.017 ± 0.002	0.038 ± 0.006	0.004 ± 0.000	0.007 ± 0.001	0.001 ± 0.000	0.004 ± 0.000	0.323 ± 0.018	2.338 ± 0.012	0.008 ± 0.001	0.001 ± 0.000
**Green Tea Powder**										
**Compound**	**Epicatechin (EC)**	**Epigallocatechin Gallate (EGCG)**			**Gallocatechin Gallate (GCG)**		**Epicatechin Gallate (ECG)**		**Total**	
Content (mg/g) ^1^	60.41 ± 0.15	35.90 ± 0.10			1.30 ± 0.01		17.04 ± 0.10		114.64 ± 0.35	

^1^ Mean of duplicate determinations.

**Table 6:** PAH contents (ng/g) in marinated pork and juice as affected by different flavorings and time length.

PAHs	0 h	2 h	4 h	8 h	12 h	24 h
marinated pork ^1,2^						
Pheneanthrene (Phe)	0.60 ± 0.01 ^e^	0.63 ± 0.05 ^e^	0.78 ± 0.04 ^d^	1.24 ± 0.16 ^c^	1.47 ± 0.04 ^b^	1.64 ± 0.09 ^a^
Pyrene (Pyr)	31.00 ± 0.15 ^f^	38.81 ± 0.72 ^e^	41.79 ± 0.79 ^d^	43.87 ± 0.85 ^c^	46.94 ± 0.98 ^b^	50.51 ± 0.64 ^a^
Benzo[ghi]perylene (BghiP)	nd ^3^	trace ^4^	0.34 ± 0.01 ^d^	0.43 ± 0.02 ^c^	0.56 ± 0.04 ^b^	0.81 ± 0.01 ^a^
Dibenz[a,h]anthracene (DBahA)	1.54 ± 0.01 ^a^	1.53 ± 0.01 ^a^	1.53 ± 0.01 ^a^	1.53 ± 0.01 ^a^	1.53 ± 0.01 ^a^	1.54 ± 0.01 ^a^
Dienzo[a,e]pyrene (DBaeP)	0.71 ± 0.01 ^a^	0.71 ± 0.00 ^a^	0.71 ± 0.00 ^a^	0.71 ± 0.00 ^a^	0.71 ± 0.00 ^a^	0.71 ± 0.01 ^a^
Dienzo[a,l]pyrene (DBalP)	0.72 ± 0.00 ^b^	0.72 ± 0.00 ^ab^	0.72 ± 0.00 ^ab^	0.72 ± 0.00 ^a^	0.72 ± 0.00 ^a^	0.72 ± 0.01 ^a^
Total	34.56 ± 0.17 ^f^	42.40 ± 0.77 ^e^	45.87 ± 0.76 ^d^	48.49 ± 0.999 ^c^	51.92 ± 0.94 ^b^	55.93 ± 0.73 ^a^
marinated pork + 0.5% cinnamon powder ^1,2^						
Pheneanthrene (Phe)	trace	trace	trace	0.44 ± 0.04 ^c^	4.83 ± 0.25 ^b^	7.29 ± 0.40 ^a^
Pyrene (Pyr)	30.61 ± 1.10 ^e^	36.08 ± 0.84 ^d^	37.15 ± 1.11 ^cd^	37.86 ± 1.01 ^bc^	39.66 ± 0.60 ^b^	45.02 ± 1.31 ^a^
Benzo[ghi]perylene (BghiP)	nd	nd	trace	0.45 ± 0.04 ^c^	1.23 ± 0.09 ^b^	3.46 ± 0.15 ^a^
Dibenz[a,h]anthracene (DBahA)	1.53 ± 0.01 ^ab^	1.53 ± 0.01 ^ab^	1.52 ± 0.00 ^ab^	1.52 ± 0.00 ^b^	1.52 ± 0.00 ^b^	1.55 ± 0.03 ^a^
Dienzo[a,e]pyrene (DBaeP)	0.71 ± 0.01 ^a^	0.71 ± 0.01 ^a^	0.71 ± 0.00 ^a^	0.71 ± 0.01 ^a^	0.71 ± 0.00 ^a^	0.72 ± 0.01 ^a^
Dienzo[a,l]pyrene (DBalP)	0.72 ± 0.01 ^ab^	0.72 ± 0.00 ^b^	0.72 ± 0.00 ^b^	0.72 ± 0.00 ^b^	0.72 ± 0.00 ^b^	0.73 ± 0.01 ^a^
Total	33.57 ± 1.09 ^e^	39.04 ± 0.86 ^de^	40.10 ± 1.14 ^cd^	41.70 ± 1.06 ^c^	48.74 ± 0.50 ^b^	58.76 ± 1.87 ^a^
marinated pork + 0.5% green tea powder ^1,2^						
Pheneanthrene (Phe)	0.33 ± 0.01 ^d^	0.78 ± 0.06 ^c^	0.79 ± 0.06 ^c^	0.84 ± 0.04 ^c^	1.05 ± 0.13 ^b^	1.77 ± 0.09 ^a^
Pyrene (Pyr)	28.80 ± 1.50 ^d^	30.94 ± 1.12 ^d^	35.05 ± 0.80 ^c^	35.22 ± 1.79 ^bc^	37.35 ± 0.82 ^b^	49.77 ± 0.76 ^a^
Benzo[ghi]perylene (BghiP)	nd	nd	nd	nd	nd	nd
Dibenz[a,h]anthracene (DBahA)	1.53 ± 0.01 ^a^	1.57 ± 0.05 ^a^	1.54 ± 0.01 ^a^	1.54 ± 0.01 ^a^	1.55 ± 0.03 ^a^	1.55 ± 0.05 ^a^
Dienzo[a,e]pyrene (DBaeP)	0.71 ± 0.00 ^c^	0.71 ± 0.00 ^c^	0.71 ± 0.00 ^c^	0.72 ± 0.01 ^bc^	0.72 ± 0.00 ^b^	0.73 ± 0.00 ^a^
Dienzo[a,l]pyrene (DBalP)	0.72 ± 0.00 ^c^	0.73 ± 0.01 ^b^	0.73 ± 0.00 ^b^	0.73 ± 0.08 ^b^	0.73 ± 0.01 ^b^	0.76 ± 0.01 ^a^
Total	32.09 ± 1.50 ^d^	34.72 ± 1.11 ^d^	38.82 ± 2.73 ^c^	39.05 ± 0.46 ^c^	41.40 ± 0.95 ^b^	54.59± 0.54 ^a^
marinated juice^1,2^						
Pheneanthrene (Phe)	0.34 ± 0.01 ^e^	0.41 ± 0.06 ^d^	0.45 ± 0.03 ^d^	0.63 ± 0.03 ^c^	0.71 ± 0.01 ^b^	0.80 ± 0.04 ^a^
Pyrene (Pyr)	23.56 ± 0.87 ^e^	23.70 ± 0.26 ^e^	26.00 ± 1.78 ^d^	33.07 ± 0.54 ^c^	37.33 ± 1.62 ^b^	40.36 ± 1.09 ^a^
Dibenz[a,h]anthracene (DBahA)	1.53 ± 0.00 ^b^	1.53 ± 0.00 ^b^	1.52 ± 0.00 ^b^	1.52 ± 0.01 ^b^	1.53 ± 0.01 ^b^	1.55 ± 0.00 ^a^
Dienzo[a,e]pyrene (DBaeP)	0.71 ± 0.00 ^c^	0.71 ± 0.00 ^c^	0.71 ± 0.00 ^bc^	0.71 ± 0.00 ^bc^	0.72 ± 0.00 ^b^	0.73 ± 0.01 ^a^
Dienzo[a,l]pyrene (DBalP)	0.72 ± 0.00 ^d^	0.72 ± 0.00 ^d^	0.72 ± 0.00 ^cd^	0.73 ± 0.00 ^bc^	0.73 ± 0.01 ^b^	0.74 ± 0.00 ^a^
Total	26.85 ± 0.86 ^e^	27.06 ± 0.24 ^e^	29.41 ± 1.75 ^d^	36.67 ± 0.53 ^c^	41.01 ± 1.62 ^b^	44.17 ± 1.05 ^a^
marinated juice + 0.5% cinnamon powder ^1,2^						
Pheneanthrene (Phe)	trace ^3^	0.38 ± 0.02 ^b^	0.42 ± 0.02 ^b^	0.70 ± 0.29 ^a^	0.77 ± 0.03 ^a^	0.85 ± 0.04 ^a^
Pyrene (Pyr)	24.19 ± 0.64 ^f^	34.96 ± 0.36 ^e^	42.76 ± 1.27 ^d^	46.41 ± 1.74 ^c^	49.13 ± 1.11 ^b^	54.52 ± 1.20 ^a^
Dibenz[a,h]anthracene (DBahA)	1.52 ± 0.00 ^b^	1.53 ± 0.00 ^b^	1.52 ± 0.00 ^b^	1.53 ± 0.01 ^b^	1.53 ± 0.01 ^b^	1.55 ± 0.01 ^a^
Dienzo[a,e]pyrene (DBaeP)	0.71 ± 0.00 ^b^	0.71 ± 0.00 ^b^	0.71 ± 0.00 ^b^	0.71 ± 0.01 ^b^	0.71 ± 0.00 ^b^	0.73 ± 0.01 ^a^
Dienzo[a,l]pyrene (DBalP)	0.72 ± 0.00 ^c^	0.72 ± 0.00 ^c^	0.73 ± 0.00 ^b^	0.73 ± 0.00 ^b^	0.73 ± 0.01 ^ab^	0.74 ± 0.00 ^a^
Total	27.14 ± 0.63 ^f^	38.29 ± 0.34 ^e^	46.14 ± 1.26 ^d^	50.06 ± 1.58 ^c^	52.87 ± 1.12 ^b^	58.39 ± 1.17 ^a^
marinated juice + 0.5% green tea powder ^1,2^						
Pheneanthrene (Phe)	0.40 ± 0.03 ^f^	0.54 ± 0.01 ^e^	0.58 ± 0.01 ^d^	0.76 ± 0.03 ^c^	0.83 ± 0.02 ^b^	0.97 ± 0.03 ^a^
Pyrene (Pyr)	22.64 ± 1.05 ^f^	26.57 ± 2.12 ^e^	31.60 ± 0.84 ^d^	37.77 ± 0.54 ^c^	40.72 ± 0.89 ^b^	47.69 ± 1.81 ^a^
Dibenz[a,h]anthracene (DBahA)	1.53 ± 0.01 ^a^	1.53 ± 0.00 ^a^	1.53 ± 0.01 ^a^	1.53 ± 0.01 ^a^	1.54 ± 0.01 ^a^	1.54 ± 0.03 ^a^
Dienzo[a,e]pyrene (DBaeP)	0.71 ± 0.00 ^c^	0.71 ± 0.00 ^c^	0.71 ± 0.00 ^bc^	0.71 ± 0.00 ^ab^	0.71 ± 0.00 ^a^	0.72 ± 0.00 ^a^
Dienzo[a,l]pyrene (DBalP)	0.72 ± 0.00 ^d^	0.73 ± 0.00 ^d^	0.73 ± 0.00 ^cd^	0.73 ± 0.00 ^bc^	0.74 ± 0.00 ^b^	0.76 ± 0.00 ^a^
Total	26.00 ± 1.09 ^f^	30.07 ± 2.11 ^e^	35.14 ± 0.83 ^d^	41.50 ± 0.56 ^c^	44.54 ± 0.87 ^b^	51.68 ± 1.81 ^a^

^1^ Data are presented as mean ± standard deviation of triplicate determinations and data with different small letters (a–f) in the same row are significantly different (*p* < 0.05); ^2^ Data with the standard deviation values <0.005 are shown as ±0.00 to maintain 2 significant digits; ^3^ not detected; ^4^ LOQ ≥ PAHs levels ≥ LOD.

**Table 7:** Changes of PAH precursor contents (ng/g) in unmarinated/marinated pork and juice as affected by different flavorings and time length.

Time Length	0 h	2 h	4 h	8 h	12 h	24 h
2-cyclohexene-1-one						
marinated pork (MP)	5.30	9.71	10.88	12.90	28.43	43.19
MP + 0.5% cinnamon powder	3.72	16.30	17.79	28.32	40.51	151.24
MP + 0.5% green tea powder	nd^1^	11.25	21.46	45.09	54.02	181.89
benzaldehyde						
marinated pork (MP)	140.93	155.59	191.86	226.28	289.67	240.47
MP + 0.5% cinnamon powder	183.88	922.47	522.05	338.95	294.31	253.05
MP + 0.5% green tea powder	200.81	121.63	177.61	155.51	176.41	180.34
trans,trans-2,4-decadienal						
marinated pork (MP)	15.06	80.45	152.74	224.86	233.75	248.04
MP + 0.5% cinnamon powder	16.35	42.03	62.44	126.85	205.47	296.07
MP + 0.5% green tea powder	25.12	40.69	48.64	71.42	123.28	218.44
Total (2-cyclohexene-1-one + benzaldehyde + trans,trans-2,4-decadienal)						
marinated pork (MP)	161.29	245.75	355.47	464.04	551.85	531.71
MP + 0.5% cinnamon powder	203.95	980.80	602.27	494.12	540.29	700.36
MP + 0.5% green tea powder	225.93	173.57	247.71	272.02	353.71	580.67
2-cyclohexene-1-one						
marinated juice (MJ)	nd	2.30	4.68	4.56	15.81	21.46
MJ + 0.5% cinnamon powder	nd	3.14	4.25	5.03	8.34	24.30
MJ + 0.5% green tea powder	nd	5.13	9.31	8.86	7.11	9.32
benzaldehyde						
marinated juice (MJ)	24.25	39.23	37.19	37.79	36.90	13.77
MJ + 0.5% cinnamon powder	30.98	45.98	53.57	51.35	40.14	36.16
MJ + 0.5% green tea powder	18.38	24.36	24.95	32.15	34.96	33.80
trans,trans-2,4-decadienal						
marinated juice (MJ)	5.92	6.10	7.22	44.38	102.00	93.32
MJ + 0.5% cinnamon powder	nd	6.43	5.16	9.84	51.90	81.73
MJ + 0.5% green tea powder	4.73	4.91	5.96	10.49	13.23	51.09
Total (2-cyclohexene-1-one + benzaldehyde + trans,trans-2,4-decadienal)						
marinated juice (MJ)	30.17	47.63	49.08	86.73	154.72	128.55
MJ + 0.5% cinnamon powder	30.98	55.55	62.98	66.22	100.38	142.20
MJ + 0.5% green tea powder	23.11	34.40	40.23	51.50	55.30	94.21

^1^ not detected.

## Data Availability

The data that support the findings of this study are available within the manuscript.

## Supplementary Materials

The following supporting information can be downloaded at: https://www.mdpi.com/article/10.3390/foods11193080/s1, Methods for determination of HA precursors (reducing sugar, amino acid, creatine/creatinine), antioxidant activity, bioactive compounds in cinnamon powder by UPLC-MS/MS, bioactive compounds in green tea powder by HPLC-DAD and PAH precursors.

## Abbreviations

List of names and abbreviations of 21 HAs and 24 PAHs used in this study.

**Abbreviation**	**Full Names**
**HAs**	
DMIP	2-amino-1,6-dimethylimidazo [4,5-b]-pyridine
IFP	2-amino-1,6-dimethyl-furo [3,2-e]imidazo [4,5-b]-pyridine
iso-IQ	2-amino-1-methyl-imidazo [4,5-f]-quinoline
IQ	2-amino-3-methyl-imidazo [4,5-f]-quinoline
MeIQ	2-amino-3,4-dimethyl-imidazo [4,5-f]- quinolin
IQ [4,5-b]	2-amino-1-methyl-imidazo [4,5-b]-quinoline
IQx	2-amino-3-methyl-imidazo [4,5-f]-quinoxaline
8-MeIQx	2-amino-3,8-dimethyl-imidazo [4,5-f]- quinoxaline
7,8-DiMeIQx	2-amino-3,7,8-dimethyl-imidazo [4,5-f]-quinoxaline
4,8-DiMeIQx	2-amino-3,4,8-dimethyl-imidazo [4,5-f]-quinoxaline
4,7,8-TriMeIQx	2-amino-3,4,7,8-dimethyl-imidazo [4,5-f]- quinoxaline (internal standard)
Phe-P-1	2-amino-5-phenylpyridine
AαC	2-amino-9 H-pyrido [2,3-b]indole
Trp-P-2	3-amino-1-methyl-5 H-pyrido [4,3-b]indole
Trp-P-1	3-amino-1,4-methyl-5 H-pyrido [4,3-b]indole
GIu-P-2	2-aminodipyrido-[1,2-a:3′,2′-d]imidazole
Glu-P-1	2-amino-6-methyldipyrido-[1,2-a:3′,2′-d]imidazole
PhIP	2-amino-1-methyl-6-phenylimidazo [4,5-b]-pyridine
Harman	1-methyl-9 H-pyrido [3,4-b]indole
Norharman	9 H-pyrido [3,4-b]indole
MeAαC	2-Amino-3-methyl-9 H-pyrido [2,3-b]indole
**PAHs**	
Nap	naphthalene
AcPy	acenaphylene
AcP	acenaphthene
Flu	fluorene
Phe	phenanthrene
Ant	anthracene
FL	fluoranthene
Pyr	pyrene
BaA	benzo[a]anthracene
CHR	chrysene
BbFL	benzo[b]fluoranthene
BaP	benzo[a]pyrene
IP	indeno [1,2,3-c,d]pyrene
DBahA	dibenzo[a,h]anthracene
BghiP	benzo[g,h,i]perylene
BjF	benzo[j]fluoranthene
BcF	benzo[c]fluorene
CcdP	cyclopenta[c,d]pyrene
MCH	5-methylchrysene
DBalP	dibenzo[a,l]pyrene
DBaeP	dibenzo[a,e]pyrene
DBaiP	dibenzo[a,i]pyrene
DBahP	dibenzo[a,h]pyrene
Triphenylene	Triphenylene (internal standard)
