# Supplementary material for: Formation and Inhibition of Heterocyclic Amines and Polycyclic Aromatic Hydrocarbons in Ground Pork during Marinating
Source: Foods. 2022 Oct 4;11(19):3080. doi: 10.3390/foods11193080 (PMC9563804; doi:10.3390/foods11193080)
Supplement: Supplementary file 1 [file foods-11-03080-s001.zip › foods-1930606-supplementary.pdf]

Supplementary Materials

for

Formation and Inhibition of Heterocyclic Amines and Polycyclic Aromatic Hydrocarbons in Ground Pork during Marinating

Yu-Wen Lai[1,†], Yu-Tsung Lee[2,†], Baskaran Stephen Inbaraj[1] and Bing-Huei Chen[1,3,*]

[1] Department of Food Science, Fu Jen Catholic University, New Taipei City 24205, Taiwan

[2] Research Center for Food and Cosmetic Safety, College of Human Ecology, Chang Gung University of Science and Technology, Taoyuan 33303, Taiwan

[3] Department of Nutrition, China Medical University, Taichung 40402, Taiwan

* Corresponding author: 002622@mail.fju.edu.tw

† These authors equally contributed to this work

Determination of HA precursors

Reducing sugar

A method based on Chen et al. [24] was used to determine reducing sugar contents in unmarinated pork, marinated pork and juice. In brief, a 0.315 g of the reagent dinitrosalicylic acid (DNS) was dissolved in 50 mL of distilled water and mixed with 10 mL of NaOH solution (0.2 g/mL), followed by adding 9.1 g of sodium potassium tartrate solution and diluting to 100 mL with distilled water. Then 1 mL was collected and mixed with 0.1 mL of various glucose standard concentrations separately, followed by heating at 100°C for 10 min, cooling to room temperature and measuring absorbance at 570 nm for preparation of the glucose standard curve and calculation of linear regression equations and R^2. Next, a 2-g pork sample was mixed with 10-mL of distilled water, after which this mixture was shaken for 60 min, centrifuged at 4000 g for 20 min and diluted to 50 mL with distilled water. Then, 0.1-mL was collected and mixed with 1 mL of DNS, followed by heating at 100°C for 10 min, cooling to room temperature and measuring absorbance at 570 nm for calculation of the glucose contents in pork and juice samples by using the linear regression equation [24].

Amino acid

The amino acid contents in unmarinated pork, marinated pork and juice was determined by a method reported by TFDA [25]. In brief, the amino acid standard solution was prepared by dissolving a 37.5-mg of each amino acid standard in 10 mL of 1 N HCl, and then a concentration range from 1.5-150 μg/mL was obtained by dilution with 0.1 N HCl. Next, a 20-μL solution was collected and mixed with 100 μL of boric acid buffer solution (0.4 M), followed by mixing thoroughly, adding 20 μL of phthaldehyde, vortexing for 60 sec, adding 20 μL of 9-fluorenylmethyl chloroformate, vortexing again

for 30 sec, adding 1280 μL of deionized water, and mixing thoroughly to obtain the derivatized amino acid standard solution. Then a 0.5 g of pork sample was mixed with 20-mL of 0.1 N HCl, after which this mixture was shaken in an ultrasonicator for 10 min, followed by diluting to 25 mL with 0.1 N HCl, filtering through a membrane filter, collecting 20 μL and mixing with 100 μL of boric acid buffer solution (0.4 M), adding 20 μL of fluorenylmethyl chloroformate, vortexing for 30 sec and adding 1280 μL of deionized water for HPLC analysis. A Poroshell HPH-C18 column (10 cm x 3.0 mm ID, particle size 2.7 μm) with flow rate at 0.5 mL/min, detection wavelength at 338 nm and 262 nm, injection volume 10 μL, column temperature 40°C and a gradient mobile phase of 40 mM NaH_2PO_4 (pH 7.8) (A) and acetonitrile/methanol/water (45:45:10, v/v/v) (B) at 93% A and 7% B initially, maintained for 0.5 min, decreased to 75% A in 5 min, 72% A in 7 min, 54% A in 12 min, 0% A in 13 min, 0% A in 16 min, raised to 93% A in 16.1 min and maintained till 20 min. The various amino acids in pork and juice samples were separated and identified through comparison of retention time and absorption spectra of unknown peaks with reference standers, followed by quantitation using a formula as described in a TFDA report [25].

Creatine and creatinine

A method based on Gibis and Loeffler [26] was used to determine creatine and creatinine contents in unmarinated pork, marinated pork and juice. Briefly, a 20-g sample was mixed with 100-mL of distilled water, after which this solution was homogenized at 24,000 rpm for 2 min, followed by standing at 18°C for 20 min, filtering through a filter paper, adding perchloric acid (1 mol/L), adding KOH to neutralize pH to 6.5 and using the assay kits to analyze creatine and creatinine in pork and juice samples.

Antioxidant activity

A method based on Serpen et al. [27] was used to determine antioxidant capacity of unmarinated pork, marinated pork and juice including DPPH and FRAP. Briefly, the DPPH (1,1-diphenyl-2-picrylhydrazyl) solution was prepared by mixing a 40-mg of the DPPH with 100 mL of ethanol and then 100 mL of deionized water was added to obtain a stock solution. For practical use, this solution was diluted with 800 mL of 50% ethanol to have an absorbance at 1.0. Next, the FRAP (ferric reduction activity potential) solution was prepared by mixing 300 mM of sodium acetate solution (pH 3.6), 10 mM of 2,4,6-Tris(2-pyridyl)-*s*-triazine (TPTZ) and 20 mM of ferric chloride solution at a ratio of 10:1:1 (v/v/v).

Then a 10 mg sample was poured into a centrifuge tube and 10-mL of DPPH or FRAP was added, followed by mixing thoroughly for 1 min, shaking at 300-400 rpm for 60 min, centrifuging at 9,200 g for 2 min, and the supernatant was collected for absorbance measurement at 525 nm for DPPH and 593 nm for FRAP. The inhibition percentage of pork and juice samples were calculated using a formula as described by Serpen et al. [27]. Furthermore, the inhibition percentage of each sample was converted to Trolox equivalent antioxidant capacity (TEAC) by preparing Trolox solutions in methanol with a concentration range from 0-600 μg/mL. Then 0.1 mL was collected and mixed with 9.9 mL of DPPH solution, after which this mixture was reacted in the dark for 30 min and the absorbance at 525 nm was measured for preparation of the standard curve and the inhibition percentage was calculated using the same formula as described by Serpen et al. [27]. As no inhibition percentage was calculated for FRAP, the absorbance at 593 nm was used for preparation of the Trolox standard curve to calculate the reducing power using a formula as described by Serpen et al. [27].

Bioactive compounds in cinnamon powder by UPLC-MS/MS

A method based on Wang et al. [28] was used to determine bioactive compounds including phenolic acid, flavonoid and cinnamaldehyde in cinnamon powder. A 0.5 g sample of cinnamon powder was mixed with 40 mL of 80% methanol and this mixture was sonicated at 60°C for 90 min, followed by centrifuging at 4,000 rpm for 15 min (25°C), collecting the supernatant, repeating extraction until the supernatant became transparent, combining the supernatants, evaporating to dryness under N_2, dissolving in 5 mL of 30% acetonitrile and filtering through a 0.22-μm membrane filter for UPLC-MS/MS analysis. A Waters ACQUITY UPLC BEH shield RP18 column (100 x 2.1 mm ID, particle size

1.7 μm) was used with flow rate at 0.3 mL/min, column temperature at 30°C and a gradient mobile phase of 0.1% formic acid solution (A) and 100% acetonitrile (B): 83% A and 17% B initially, increased to 20% B in 1 min, maintained for 4 min, and increased to 100% B in 7 min, maintained for 2 min. The multiple reaction monitoring (MRM) mode was used for detection with the spray voltage at 3000 V, sweep gas flow rate at 0 arbitrary units, sheath gas flow rate at 38 arbitrary units, auxiliary gas flow rate at 12 arbitrary units, ion transfer tube temperature at 329°C and vaporizer temperature at 279°C. Quantitation of the various bioactive compounds in cinnamon powder was carried out using an external standard method [28].

Bioactive compounds in green tea powder by HPLC-DAD

A method based on Lin et al. [29] was used to determine bioactive compounds including epicatechin (EC), epigallocatechin gallate (EGCG), gallocatechin gallate (GCG) and epicatechin gallate (ECG) in green tea powder. Briefly, 0.2-g sample of green tea powder was mixed with 4 mL of 50% ethanol and this mixture was sonicated at room temperature for 60 min, followed by centrifuging at 4,000 rpm for 30 min (25°C), collecting the supernatant and filtering through a 0.22-μm membrane filter for HPLC-DAD analysis. A Gemini C18 column (250 x 4.6 mm I.D., particle size 5 μm) was used with flow rate at 1 mL/min, column temperature at 30°C and a gradient mobile phase of 0.1% formic acid solution (A) and 100% acetonitrile (B): 88% A and 12% B initially, maintained for 3 min, increased to 20% B in 6 min, 25% B in 3 min, and maintained for 13 min. Identification and quantitation of EC, EGCG, GCG and ECG was performed using the same method as described by Lin et al. [29].

Determination of PAH precursors

A method based on Bueno et al. [30] was used to analyze PAH precursors by using a SPME coupled with GC-MS. The PAH precursor standards including 2-cyclohexene-1-one, benzaldehyde and trans,trans-2,4-decadienal were purchased from Sigma-Aldrich (St Louis, USA). Stock solutions of these standards (2000 μg/mL each) were prepared in methanol and stored at -20°C until use. Then the stock solutions were further diluted to 50 μg/mL as working solution.

A 0.5-g sample was transferred into a 20-mL headspace vial, and 2.5 mL of water was added. Then the sample was preheated for 10 min at 65°C. The fiber head (65 um PDMS/DVB, Supelco., PA, USA) for solid phase microextraction (SPME) was inserted into the sample vial, and the fiber head was exposed to the headspace for 20 min at 65°C. Following extraction, the fiber was inserted into the GC-MS inlet for further analysis. A gas chromatograph (Agilent 7890B, CA, USA) -mass spectrometer (Agilent 5977A, CA, USA) equipped with an autosampler and headspace sampling unit was used. An HP-5MS column (30 m × 0.25 mm × 0.25 um film thickness, Agilent, CA, USA) was used for separation of PAH precursors. The fiber was desorbed and maintained in the injection port (260°C) for 1 min with a splitless mode. The carrier gas was helium with a flow rate at 1 mL/min. The initial column temperature was 40°C, maintained for 4 min, increased to 50°C at 5°C/min and held for 2 min, raised to 120°C at 5°C /min, held for 3 min, finally increased to 260°C at 30°C/min, maintained for 5 min. The mass spectrometer was operated in the electron-ionization (EI) mode at an ionization voltage of 70 eV. The SIM mode was used for identification and quantitation of PAH precursors including 2-cyclohexene-1-one, benzaldehyde and trans,trans-2,4-decadienal at m/z 68, 105 and 81, respectively. All the MS data were analyzed by a MassHunter software. The concentrations of the PAH precursors were expressed as ng/g and determined using the standard calibration curves of the 3 precursors including 2-cyclohexene-1-one (0.1-20 ng/mL), benzaldehyde (0.1-800 ng/mL) and trans,trans-2,4-decadienal (4-100 ng/mL).

References

24. Chen, Y.; Chien, J.; Inbaraj, B.S.; Chen, B.H. Formation and inhibition of cholesterol oxidation products during marinating of pig feet. *J Agric. Food Chem.* **2012**, *60*, 173-179.
25. TFDA, Taiwan Food and Drug Administration. Method of test for free amino acids, glucosamine and taurine in foods, **2017**.

26. Gibis, M.; Loeffler, M. Effect of creatine and glucose on formation of heterocyclic amines in grilled chicken breasts. *Foods*, **2019**, *8*, 616.
27. Serpen, A.; Gokmen, V.; Fogliano, V. Total antioxidant capacities of raw and cooked meats. *Meat Sci*. **2012**, *90*, 60-5.
28. Wang, F.; Pu, C.; Zhou, P.; Wang, P.; Liang, D.; Wang, Q.; Hu, Y.; Li, B.; Hao, X. Cinnamaldehyde prevents endothelial dysfunction induced by high glucose by activating Nrf2. *Cell. Physiol. Biochem.* **2015**, *36*, 315-324.
29. Lin, Y.H.; Wang, C.C.; Lin, Y.H.; Chen, B.H. Preparation of catechin nanoemulsion from oolong tea leaf waste and its inhibition of prostate cancer cells DU-145 and tumors in mice. *Molecules* **2021**, *26*, 3260.
30. Bueno, M.; Resconi, V.C.; Campo, M.M.; Ferreira, V.; Escudero, A. Development of a robust HS-SPME-GC-MS method for the analysis of solid food samples. Analysis of volatile compounds in fresh raw beef of differing lipid oxidation degrees. *Food Chem.* **2019**, *281*, 49-56.
